# “Clickable” Polymer Brush Interfaces: Tailoring Monovalent to Multivalent Ligand Display for Protein Immobilization and Sensing

**DOI:** 10.1021/acs.bioconjchem.2c00298

**Published:** 2022-08-22

**Authors:** Aysun Degirmenci, Gizem Yeter Bas, Rana Sanyal, Amitav Sanyal

**Affiliations:** † Department of Chemistry, Bogazici University, Istanbul 34342, Turkey; ‡ Center for Life Sciences and Technologies, Bogazici University, Istanbul 34342, Turkey

## Abstract

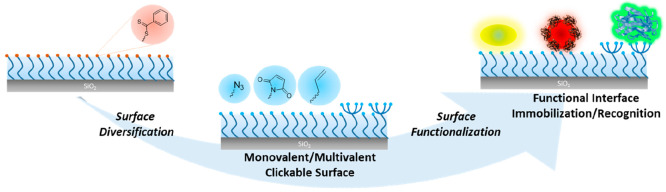

Facile and effective functionalization of the interface of polymer-coated surfaces allows one to dictate the interaction of the underlying material with the chemical and biological analytes in its environment. Herein, we outline a modular approach that would enable installing a variety of “clickable” handles onto the surface of polymer brushes, enabling facile conjugation of various ligands to obtain functional interfaces. To this end, hydrophilic anti-biofouling poly(ethylene glycol)-based polymer brushes are fabricated on glass-like silicon oxide surfaces using reversible addition–fragmentation chain transfer (RAFT) polymerization. The dithioester group at the chain-end of the polymer brushes enabled the installation of azide, maleimide, and terminal alkene functional groups, using a post-polymerization radical exchange reaction with appropriately functionalized azo-containing molecules. Thus, modified polymer brushes underwent facile conjugation of alkyne or thiol-containing dyes and ligands using alkyne–azide cycloaddition, Michael addition, and radical thiol–ene conjugation, respectively. Moreover, we demonstrate that the radical exchange approach also enables the installation of multivalent motifs using dendritic azo-containing molecules. Terminal alkene groups containing dendrons amenable to functionalization with thiol-containing molecules using the radical thiol–ene reaction were installed at the interface and subsequently functionalized with mannose ligands to enable sensing of the Concanavalin A lectin.

## Introduction

The past decade has witnessed the evolution of polymeric surface coatings from a simple protection barrier to a functional interface, which imparts functional attributes to the material through specific interactions and communication with its environment. In particular, polymeric coatings bearing bioactive ligands ranging from small molecules to biomacromolecules play a critical role in realizing various diagnostic and biosensing platforms.^1−5^ For such applications, a polymeric coating that is stable in an aqueous environment, inherently anti-biofouling, and can be easily conjugated with biological probes is often desirable. In light of the demand for workability under aqueous conditions, as a general approach, hydrophilic polymers are chemically tethered onto the underlying inorganic substrate through either a “graft-to” or “graft-from” approach. Due to widespread applications of functional surfaces, polymer brushes have emerged as an attractive coating platform due to their versatile nature.^6−9^ The “graft-from” approach entails the growth of polymer chains directly from surfaces immobilized with polymerization initiators or chain transfer agents (CTAs).^10,11^ Employment of contemporary controlled/living radical polymerization techniques allows control over their thickness and architecture. Also, these techniques furnish coatings with diverse chemical compositions due to the high tolerance of recent polymerization techniques toward a wide variety of functional groups. Furthermore, effective post-polymerization modification of polymeric materials can be undertaken using reactions from the “click” chemistry toolbox.^12−16^ In this regard, the introduction of various “clickable” functional groups in polymer brushes has been exploited for their modification using diverse “click” reactions. Commonly used transformations include the Huisgen-type copper-catalyzed [3 + 2] azide–alkyne cycloaddition^17−19^ and metal-free “click” reactions such as thiol–ene,^20^ thiol–yne,^21,22^ thiol–maleimide,^23,24^ and Diels–Alder cycloaddition.^25^ In particular, methods such as nitroxide-mediated polymerization (NMP),^26−29^ atom-transfer radical polymerization (ATRP),^30−33^ and reversible addition–fragmentation chain transfer (RAFT) polymerization^34−37^ have been widely used for obtaining polymer brushes. There is an increasing interest in employing RAFT polymerization to fabricate polymeric brushes due to its metal-catalyst free nature, high compatibility with various functional groups, and efficient polymerization at moderate temperatures with a high level of oxygen tolerance.

While clickable handles can be introduced as side-chain residues or at the chain end, in a dense surface tethered polymer brush, it is the latter position at the top of the surface that mainly interacts with the external environment. These positions act as the location of choice for installing bioactive functional molecules that can enable specific recognition of proteins, cells, and bacteria. Thus, a facile methodology to install various reactive groups at the chain end of polymer brushes will enable one to readily obtain such functional interfaces. RAFT polymerization in this context appears to be a suitable candidate since, apart from advantages like its metal-free nature, the chain end of polymers obtained using this method bears the thioester or trithio-carbonate groups, which can undergo a variety of chemical transformations.^38−40^ Azo group containing molecules which decompose through either thermal or photochemical activation furnish radical intermediates which can act as polymerization initiators,^41^ fragmentation units,^42^ or chain-terminating agents for polymers obtained through RAFT polymerization.^43−46^

**Scheme 1:**
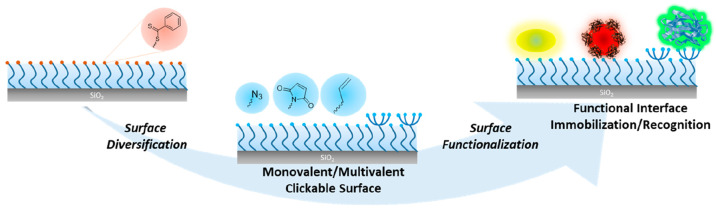
Illustration of Diversification of Surface Functionality of Polymer Brushes for Fabrication of Functional Interfaces

Herein, we demonstrate that polymer brushes fabricated on a Si/SiO_2_ glass-like surface using RAFT polymerization are amenable to the facile transformation of their thioester-based chain end group into a variety of monovalent or multivalent reactive clickable groups. These clickable handles can be subsequently conjugated with various functional molecules ranging from fluorescent dyes to bioactive ligands for biomolecular immobilization and sensing (Scheme 1). In particular, poly(ethylene glycol)-based polymer brushes were obtained using surface-initiated RAFT polymerization (SI-RAFT), followed by a radical-exchange reaction with azo-containing molecules for their end groups transformation. Azide, maleimide, and alkene groups are installed at the brush interface for subsequent functionalization using the azide–alkyne, thiol-maleimide, and radical thiol–ene click reactions, respectively. Effective conjugation of fluorescent dyes and bioactive ligands such as biotin and mannose are demonstrated. Additionally, the method uses dendritic molecules to install clustered multivalent functional groups at the interface. Functionalization of obtained dendritic structures at the surface is used to display mannose, a motif specific for recognition of Concanavalin A (ConA). The advantage of installing such multivalent ligand clusters is demonstrated by efficient sensing of target lectin.

## Results and Discussion

### Preparation and Characterization of Thioester-Containing Parent Polymer Brushes

First, a hydrophilic polymer brush containing oligo(ethylene glycol) groups was synthesized using SI-RAFT polymerization on Si/SiO_2_ surfaces. Immobilization of the RAFT CTA onto the glass-like inorganic surface was undertaken using a silyltriethoxy group-containing phenylthioester molecule.^47^ Briefly, a clean Si/SiO_2_ surface was incubated in a toluene solution containing the surface reactive CTA under a nitrogen blanket. After a specified time, the surface was rinsed with toluene to remove any unbound reagents and dried under a nitrogen stream. Successful immobilization of the RAFT CTA on the Si/SiO_2_ surface was confirmed using FTIR-ATR spectroscopy and a change in water contact angle (an increase from 3° to 63°). Before the polymerization, the RAFT CTA was micropatterned on the wafer surface using UV exposure through a photomask. Photoirradiation leads to decomposition of RAFT initiator in the exposed areas, and subsequently, SI-RAFT of DEGMA only takes place on the nonirradiated surface. This patterned growth enables facile height analysis of the polymer brush thickness using atomic force microscopy (AFM).^48^

**Figure 1:**
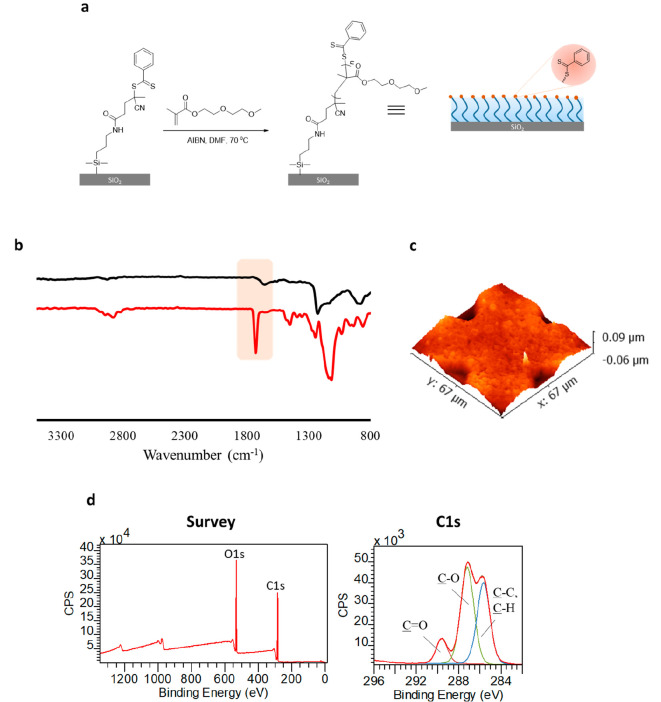
(a) Fabrication of DEGMA polymer brushes, (b) FTIR spectra of CTA (black line) and polymer brush-coated surface (red line), (c) AFM cross section of a polymer brush, and (d) XPS analysis plots of a polymer brush-coated surface.

As the first step, diethylene glycol methacrylate (DEGMA) was utilized as a monomer to obtain hydrophilic polymer brushes with anti-biofouling characteristics. Polymerization was carried out by immersing the CTA-functionalized micropatterned surface at 70 °C in a DMF solution containing DEGMA and azo-isobutyronitrile (AIBN) as an initiator under a nitrogen atmosphere (Figure 1a). First, the formation of a polymer brush coating on the Si/SiO_2_ surface was confirmed by Fourier transform infrared attenuated total reflectance (FTIR-ATR) analysis. The FTIR spectrum showed the expected C=O and C–O stretching vibrations at 1727 and 1110 cm^–1^, respectively (Figure 1b, red line). The chemical structure of the DEGMA-based brush was also investigated by X-ray photoelectron spectroscopy (XPS) analysis. The XPS survey scan revealed C 1s and O 1s signals at 285.0 and 533.0 eV, respectively (Figure 1d). The C 1s high resolution scan survey could be deconvoluted into three Gaussians with the expected relative areas for three carbon atoms at 288.9, 286.5, and 285.0 eV C=O, C–O–C/C–S/ C–N, and C–C, respectively (Figure 1d). The O 1s high-resolution scan survey could be deconvoluted into two Gaussians at 533.7 and 532.7 eV due to O=C and C–O groups, respectively (Figure S11). DEGMA polymer brush thickness was determined as 52 ± 2 nm, using AFM evaluation of step heights of the cross-sectional profile of micropatterned surface (Figure 1c, Figure S12). The correlation of brush height with polymerization time indicated that the thickness of the polymeric coating could be controlled by the extent of polymerization (Figure S13). The grafting density (σ, chains/nm^2^) of DEGMA polymer brush was calculated as 0.64 from AFM thickness (*h*, nm) and number-average molecular weight (*M*_n_, g/mol) of free polymers in solution (see the Supporting Information for details).

### Installation of “Clickable” Functional Group at the Interface

#### Azide Functional Group

**Figure 2:**
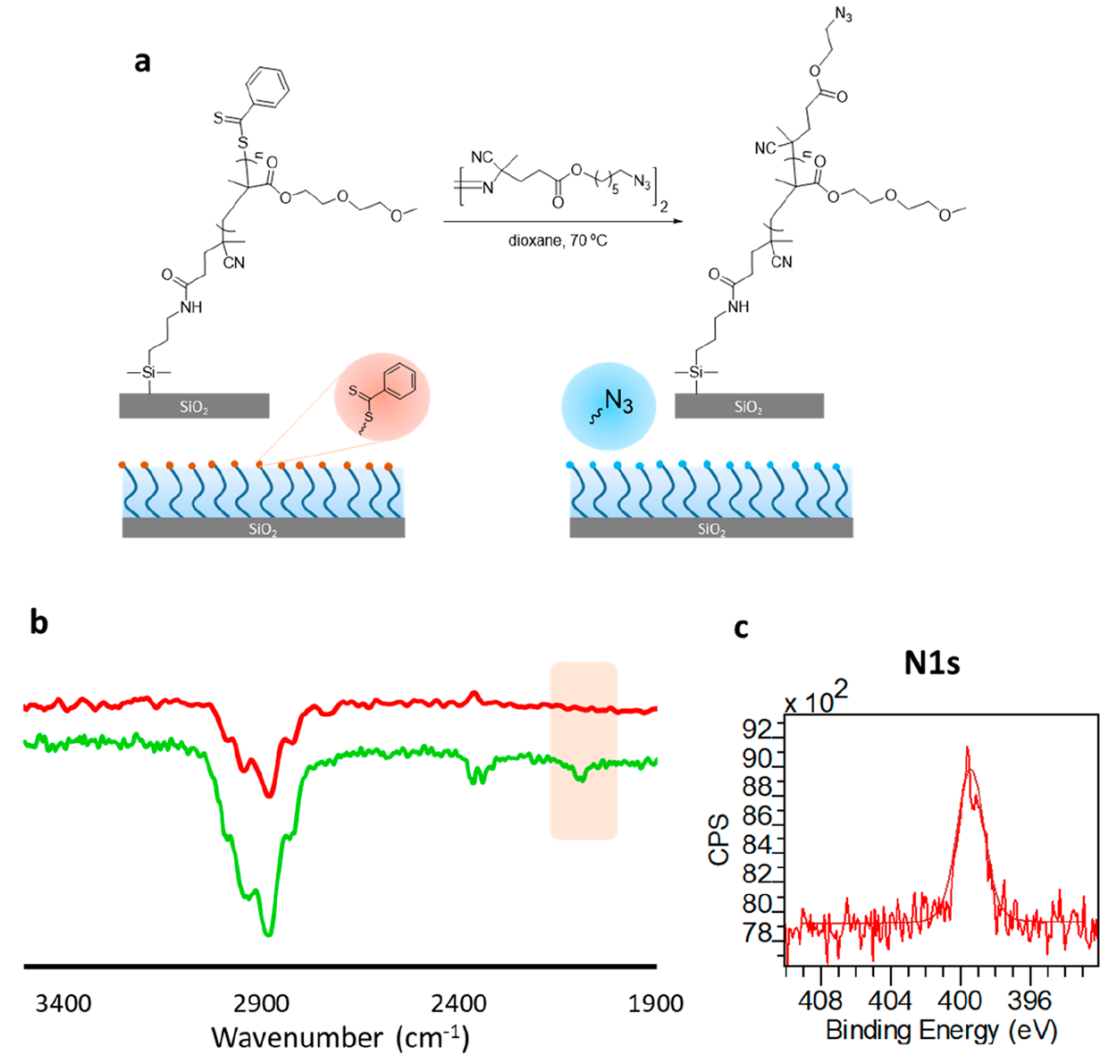
(a) Introduction of “clickable” azide groups on polymer brush surface, (b) FTIR spectra of DEGMA-containing brush (red line) and azide functionalized DEGMA-containing brush (green line), and (c) the high-resolution XPS N 1s spectrum of azide functionalized DEGMA-containing brush.

End-group functionalization of the DEGMA-containing brush was carried out with azobis-azide (azobis-N_3_) under a nitrogen atmosphere for 24 h (Figure 2a). Successful attachment of azide groups on brushes was confirmed using XPS and FTIR analysis. The C 1s and O 1s high-resolution peaks could be satisfactorily deconvoluted to account for expected carbon and oxygen atoms (Figure S14). Notably, the characteristic N atom peak on the azide (N_3_) modified surface was visible at 398.5 eV in the high-resolution XPS N 1s spectra of the DEGMA polymer brush, presumably arising from the nitrogen atoms in the newly added azide and cyano groups (Figure 2c). In addition, the FTIR spectrum of the azide-modified surface displayed the expected −N_3_, C=O, and C–O stretching vibrations at 2095, 1727, and 1116 cm^–1^, respectively (Figure 2b, green line). Azide-terminated brush thickness was determined as 51 ± 3 nm using AFM, which is similar to that of the parent brush, thus indicating no degradation of polymer brush during the end group exchange process.

#### Maleimide Functional Group

**Figure 3:**
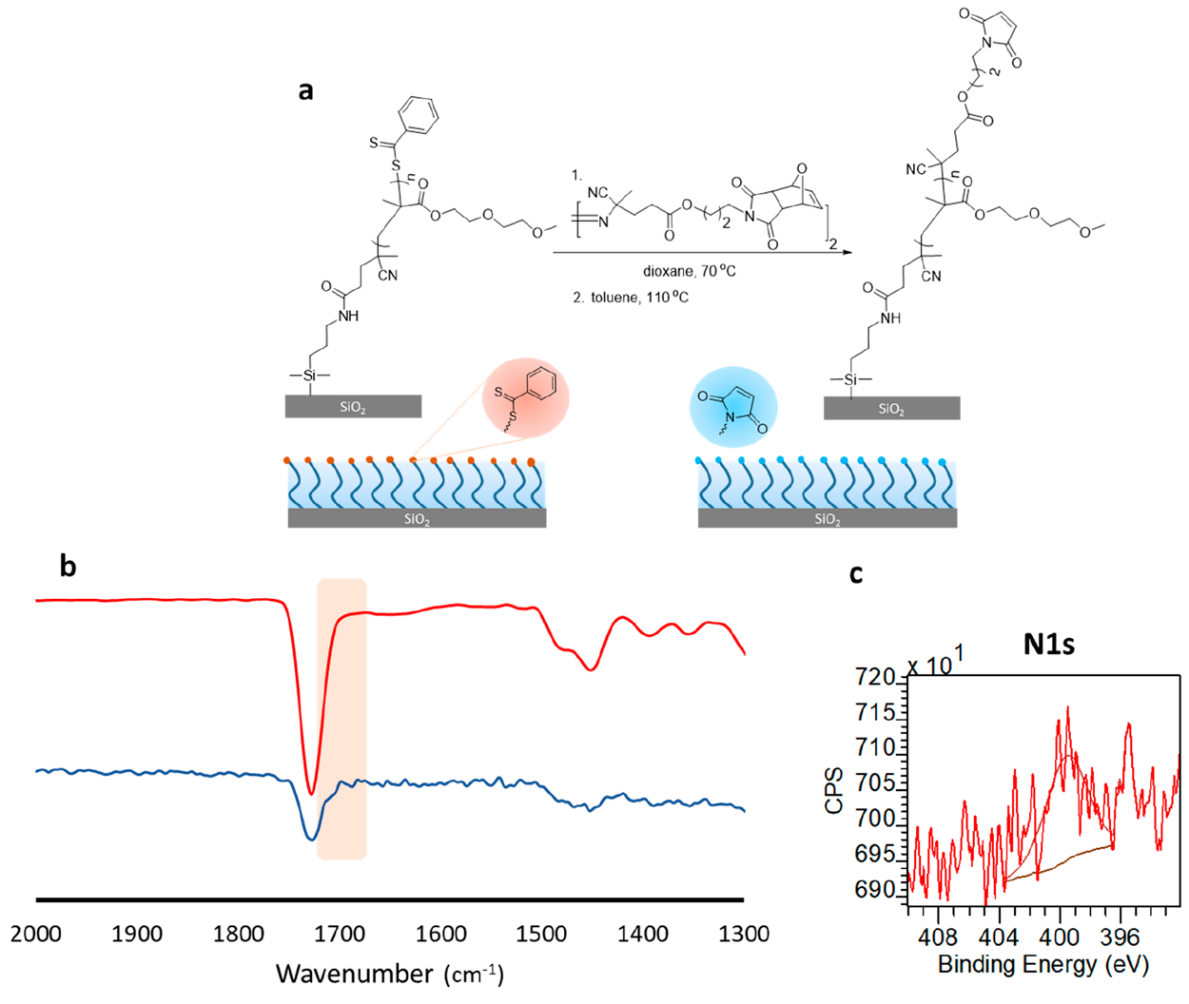
(a) Fabrication of maleimide-containing polymer brush, (b) FTIR spectra of maleimide-terminated polymer brushes (blue line) compared with parent polymer brush (red line), and (c) the high-resolution XPS scan of N 1s.

To obtain thiol-reactive maleimide functional groups on polymer brush surface, first, masked-maleimide-group-terminated DEGMA polymer brushes were prepared using an azo-containing furan-protected maleimide using the radical cross-coupling approach (Figure 3a). The successful end group modification of brushes was confirmed using XPS and FTIR spectroscopy. The C 1s high resolution scan survey could be deconvoluted into three Gaussians with the expected relative areas for three carbon atoms at 288.5, 286.1, and 285.0 eV for C=O, C–O–C, and C–C, respectively, and the O 1s high-resolution scan survey could be deconvoluted into two Gaussians at 533.5 and 532.5 eV for the O=C and C–O groups, respectively (Figure S15). The N 1s high-resolution scan survey may be deconvoluted into one Gaussian at 400.0 eV due to the amide (O=C–N) group (Figure S15). Investigation of the FTIR spectrum of the furan-protected maleimide-terminated surface indicated a new carbonyl peak at 1698 cm^–1^ belonging to the masked maleimide group. Consequently, the FTIR spectrum showed two C=O and one C–O stretching vibrations at 1727, 1698, and 1110 cm^–1^, respectively. In the next step, the polymer brushes were activated using the retro Diels–Alder reaction by removing the furan groups by heating the polymer brushes at 110 °C for 4 h. The unmasking of the maleimide group was confirmed using XPS and FTIR spectroscopy. The C 1s high resolution scan survey could be deconvoluted into three Gaussians with the expected relative areas for three carbon atoms at 288.8, 286.4, and 285.0 eV for C=O, C–O–C, and C–C, respectively (Figure S16). The O 1s high-resolution scan survey could be deconvoluted into two Gaussians at 533.6 and 532.6 eV due to the O=C and C–O groups, respectively (Figure S16). The N 1s high-resolution scan survey was fitted with a Gaussian at 399.7 eV due to the amide (O=C–N) group (Figure 3c). Notably, the characteristic peak of the protected maleimide carbonyl group at 1700 cm^–1^ shifted to 1704 cm^–1^ after deprotection of the furan groups, similar to previous reports^23^ (Figure 3b, blue line). The thickness of both the furan-protected maleimide-terminated polymer brush and the maleimide-terminated brush was 52 ± 2 nm using AFM, thus suggesting no detrimental effect of the thermal activation step on the polymer chains.

#### Terminal-Alkene Functional Group

**Figure 4:**
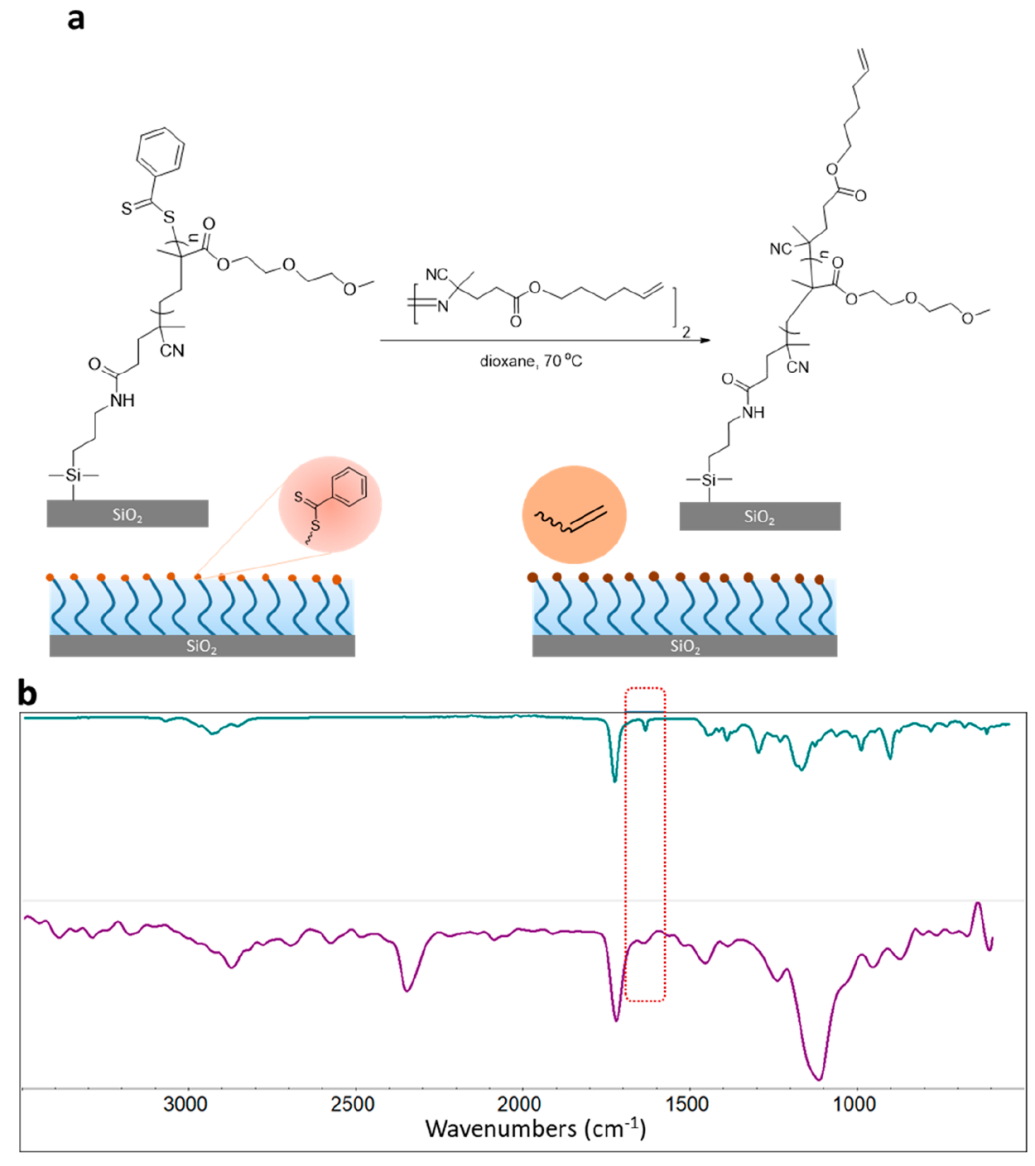
(a) Fabrication of alkene-containing polymer brush and (b) FTIR spectra of azobis-G0-ene (mint green line) and alkene (azobis-G0-ene) functionalized DEGMA-containing brush (purple line).

To obtain thiol-reactive inactivated alkene functional groups as end chains on polymer brushes, a DEGMA-containing brush was functionalized with azobis-G0-ene under a nitrogen atmosphere for 24 h (Figure 4a). The successful end group modification of brushes was confirmed using XPS and FTIR spectroscopy. The C 1s XPS high resolution scan survey could be deconvoluted into three Gaussians with the expected relative areas for three carbon atoms at 288.9, 286.5, and 285.0 eV for C–O, C–O–C, and C–C, respectively (Figure S17). Likewise, the O 1s high-resolution scan survey could be deconvoluted into expected Gaussians (Figure S17). Additionally, a comparison of the FTIR spectra of a pure azobis-G0-ene small molecule (mint green line) and azobis-G0-ene (alkene) functionalized DEGMA brush (purple line) indicated the presence of the C=C stretching on the polymer brush at 1642 cm^–1^, which implied the successful installation of the alkene unit (Figure 4b).

### Functionalization of “Clickable” Interfaces

#### Functionalization of Azide-Containing Brushes

**Figure 5:**
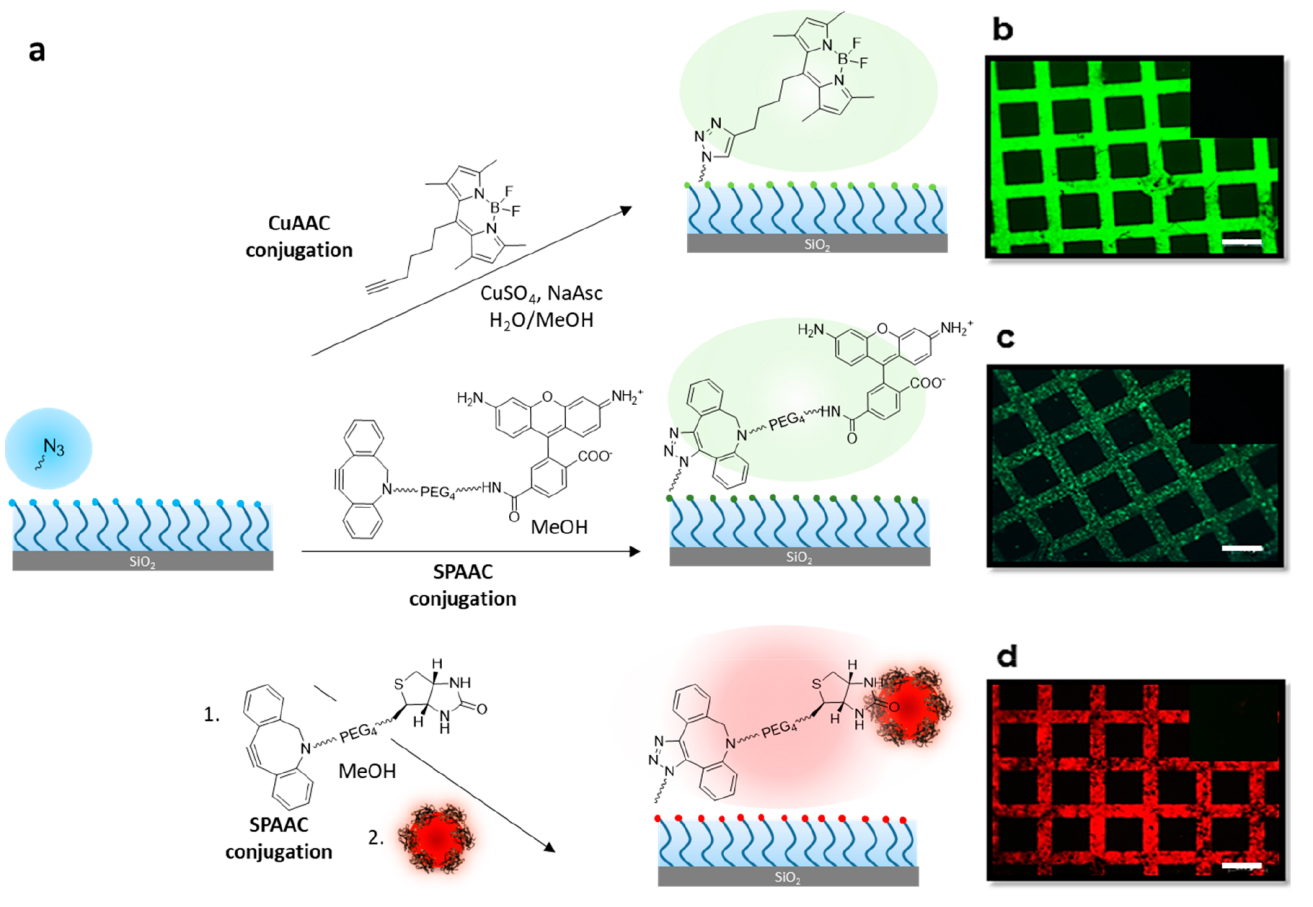
(a) Modification of azide-terminated polymer brushes with BODIPY-alkyne, DBCO-carboxyrhodamine, and DIBO-biotin followed by streptavidin-coated Qdot nanoparticles; fluorescence microscopy images of a micropatterned azide-terminated polymer brush after modification with (b) BODIPY-alkyne, (c) DBCO-carboxyrhodamine, and (d) DIBO-biotin/streptavidin-coated Qdot nanoparticles (scale bar is 100 μm). The insets show a lack of fluorescence in control experiments.

Azide group-containing polymer brushes were functionalized using Cu-catalyzed and Cu-free SPAAC cycloaddition reactions (Figure 5). We investigated functionalization using both methods since both approaches present advantages and disadvantages. While the Cu-catalyzed reaction may lead to residual amounts of a metal impurity, the materials utilized are readily available and inexpensive. The SPAAC reaction, on the other hand, proceeds without any metal catalyst, but the cyclo-octyne reactive handle involves multistep synthesis and is expensive. First, a post-polymerization modification of the azide-containing brush was carried out using a BODIPY-alkyne dye via the Huisgen 1,3-dipolar cycloaddition. After rinsing the dye-modified surface using copious amounts of organic solvent, brushes were characterized using FTIR spectroscopy. After modification with BODIPY-alkyne, the azide (N_3_) signal at 2095 cm^–1^ disappeared (Figure S25, orange line). Additionally, the fluorescence microscopy image in Figure 5b also suggests the successful attachment of BODIPY-dye because of its typical bright green fluorescence.

As an alternative, modification of the azide-containing surface was carried out using the SPAAC reaction in the presence of DBCO-PEG_4_-carboxyrhodamine. As observed for the Cu-catalyzed reaction, after modification with DBCO-carboxyrhodamine, the azide group (N_3_) signal at 2095 cm^–1^ disappeared (Figure S25, black line). Additionally, conjugation of carboxyrhodamine dye was evident from the presence of its characteristic green fluorescence (Figure 5c). As a control, parent polymer brushes devoid of the azide group were treated with respective dyes in Cu-catalyzed and SPAAC reaction-based functionalization. As expected, no fluorescence was observed for these surfaces.

To probe the efficiency of these azide-terminated polymer brushes for attachment of bioactive ligands at the interface, conjugation of a DBCO-containing biotin ligand was investigated. Surfaces conjugated with bioactive ligands either can be used to detect the presence of a target analyte such as specific proteins or can be used to immobilize biomolecules for specific applications. An azide-terminated patterned polymer brush was treated with a solution of DIBO-biotin. After rinsing off any unbound biotin ligands by aqueous wash, obtained biotinylated surfaces were treated with a solution containing streptavidin-coated CdSe quantum dots. After modification, the fluorescence microscopy image in Figure 5d shows the successful attachment of streptavidin-coated CdSe quantum dots nanoparticles. DEGMA brushes devoid of the terminal azide group were used as the control surface. They were treated with DIBO-biotin and streptavidin-coated CdSe quantum dots under the same conditions to confirm the absence of nonspecific attachment.

#### Functionalization of Maleimide-Containing Brushes

**Figure 6:**
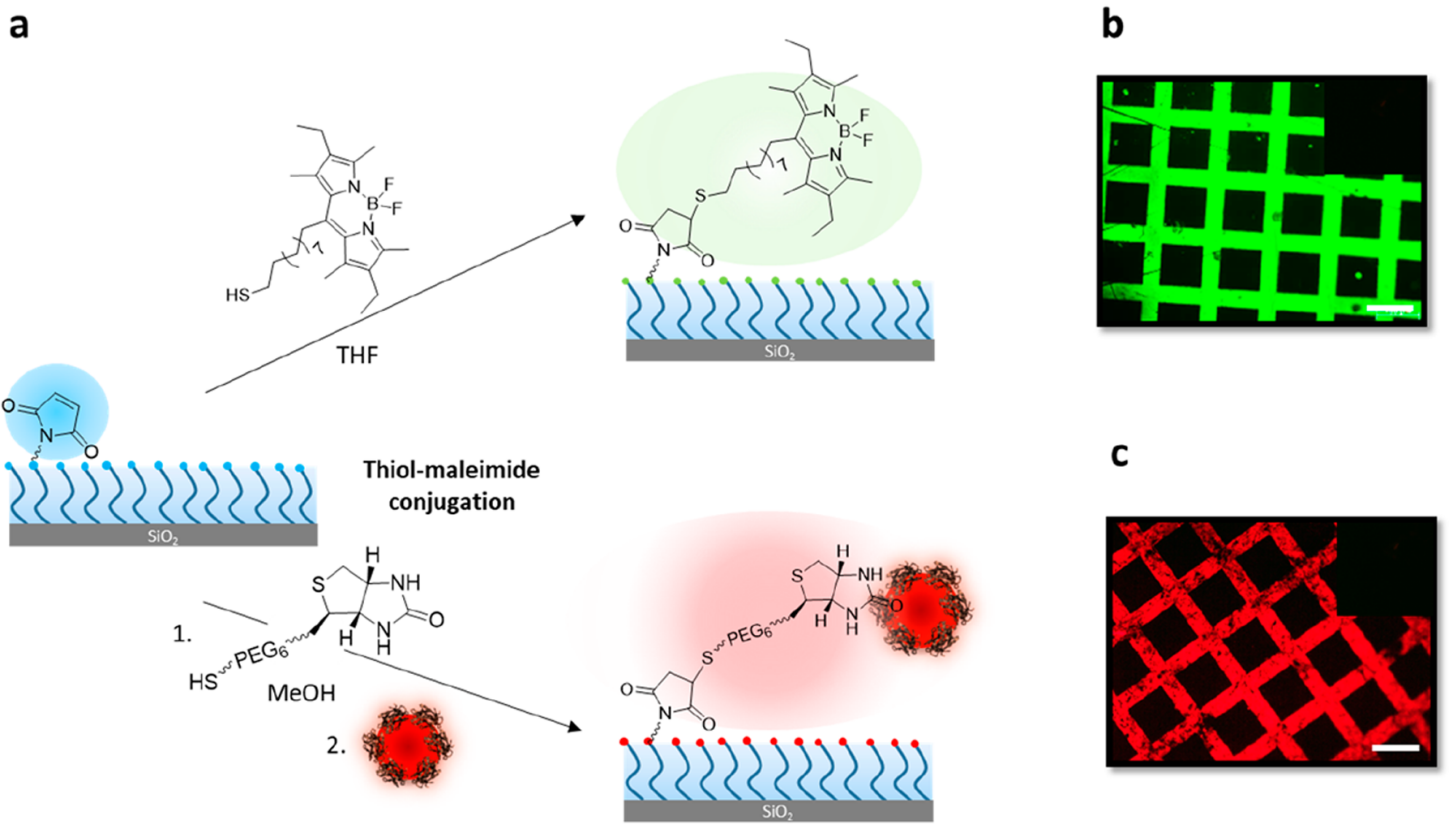
(a) Post-polymerization modification of maleimide-terminated DEGMA polymer brushes with BODIPY-SH as well as Biotin-SH/streptavidin-coated Qdot nanoparticles; fluorescence image after modification with (b) BODIPY-SH, and (c) Biotin-SH/streptavidin-coated Qdot nanoparticles (scale bar is 100 μm). The insets show a lack of fluorescence in control experiments.

The electron deficient alkene group in the maleimide moiety is known to undergo efficient conjugate addition with thiol-containing nucleophiles under mild conditions, and has been extensively used for functionalization of polymeric coatings.^49^ The first post-polymerization modification of the maleimide-terminated brush was undertaken using Michael addition of a thiol-containing fluorescent dye, namely, BODIPY-SH (Figure 6a). Since the dye is hydrophobic, the polymer brush-coated surface was immersed in a solution of BODIPY-SH in THF. A furan-protected maleimide-terminated polymer brush was used as a control surface and treated with BODIPY-SH under the same conditions. After rinsing off any physically adhering dye on the brushes using organic solvents, samples were examined using fluorescence microscopy. While the typical bright green fluorescence due to the BODIPY moiety was evident on the maleimide-containing brushes (Figure 6b), thus suggesting successful conjugation, the masked maleimide-group-containing brushes did not show any observable fluorescence (Figure 6b inset). We then utilized the thiol–maleimide conjugation to install biotin, a bioactive ligand (Figure 6c). The polymer brush was treated with biotinylated hexa(ethylene glycol)undecanethiol (Biotin-SH) in methanol. After rinsing off any unbound biotin, the biotinylated polymeric interface was incubated in a solution of streptavidin-coated quantum dots. As a control experiment, the thioester-terminated parent polymer brush, devoid of any maleimide groups, was also treated with a solution of biotin-thiol and streptavidin-coated CdSe nanoparticles under the same conditions. Upon analysis of thus treated surfaces with fluorescence microscopy, successful immobilization of streptavidin-coated nanoparticles was evident from the presence of red fluorescence (Figure 6c). On the other hand, the polymer brush-coated surface in the control experiment did not show any noticeable fluorescence, thus suggesting specific immobilization was directed by the presence of the ligand (Figure 6c inset).

#### Functionalization of Alkene-Containing Brushes

**Figure 7:**
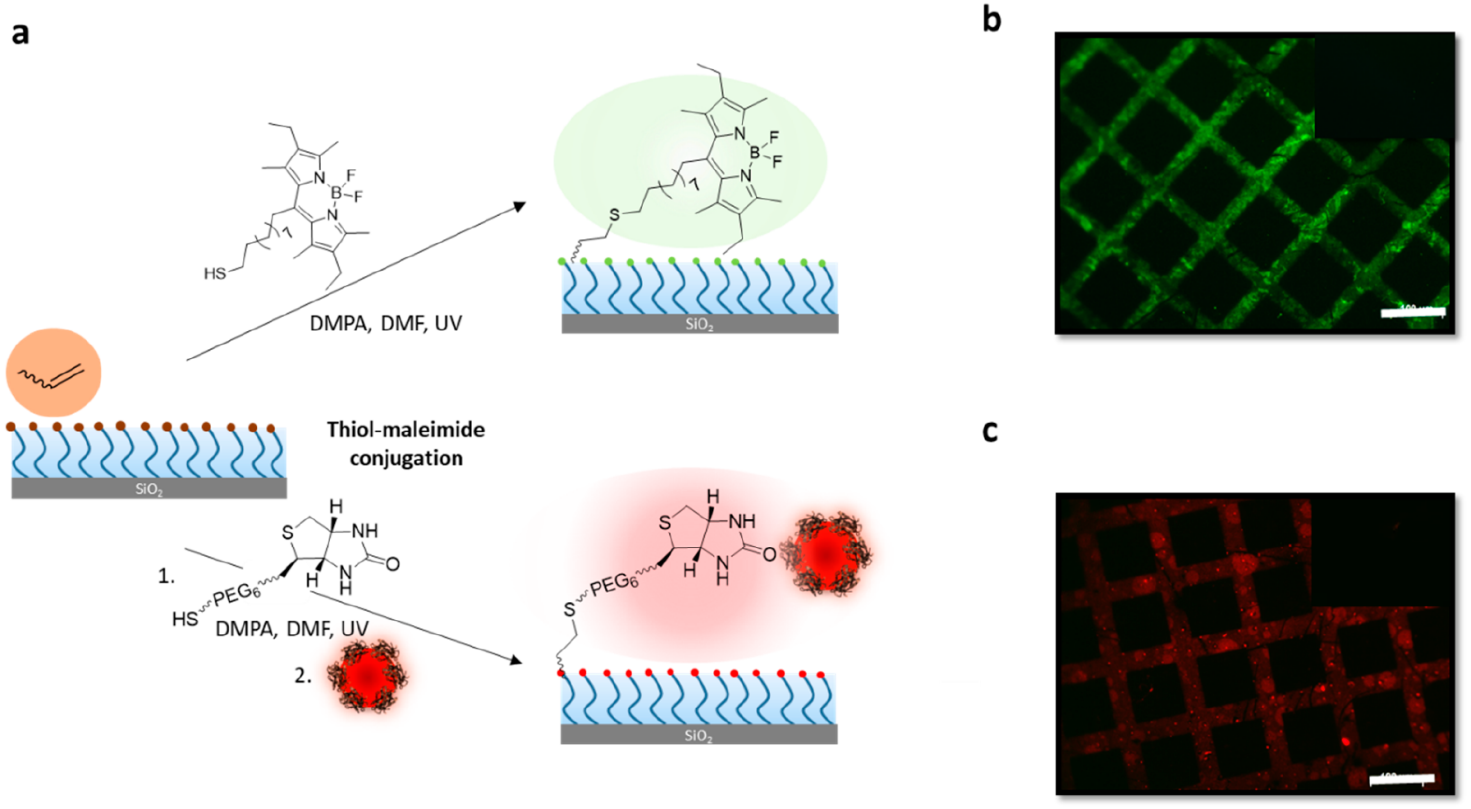
(a) Post-polymerization modification of alkene-terminated DEGMA polymer brushes with BODIPY-SH as well as Biotin-SH/streptavidin-coated Qdot nanoparticles, (b) fluorescence image of a micropatterned alkene-terminated DEGMA polymer brush after modification with BODIPY-SH, and (c) fluorescence image of a micropatterned alkene-terminated DEGMA polymer brush after modification with Biotin-SH/streptavidin-coated Qdot nanoparticles (scale bar is 100 μm). The insets show a lack of fluorescence in control experiments.

The alkene functional group presenting polymer brushes is amenable to modification using the radical thiol–ene “click” reaction. In this regard, a photochemical radical thiol–ene addition of a thiol-containing BODIPY dye was investigated. The polymer-brush-coated surface was immersed in a solution containing the thiol-containing dye and DMPA as a photoinitiator, followed by exposure to UV irradiation. As a control reaction, thioester-terminated DEGMA-containing brush was utilized and treated with thiolated dye under similar conditions. After the functionalization, surfaces were washed with organic solvents to remove any unbound reagents, followed by their examination using fluorescence microscopy. The green fluorescence suggested successful conjugation of the thiol-containing dye to the alkene-containing brushes (Figure 7b). As expected, the control surface with DEGMA-containing brushes did not display any significant fluorescence (Figure 7b inset). Like the previous surface functionalizations, conjugation of biotin using thiol–ene chemistry was also examined. Alkene end-group-terminated brushes were conjugated with Biotin-SH in the presence of DMPA under UV light. As a control surface, thioester-terminated DEGMA-containing brushes were used and treated with Biotin-SH under the same conditions. Thus, treated surfaces were washed with copious amounts of organic solvents to remove any unbound biotin. After that, the biotinylated and control polymer brushes were incubated in a solution of streptavidin-coated quantum dots. The appearance of red fluorescence indicated successful immobilization of streptavidin-coated nanoparticles onto the biotinylated polymer brushes (Figure 7c). Moreover, no observable fluorescence on the thioester-terminated control polymer brushes (Figure 7c inset).

### Preparation and Characterization of Multivalent Dendron-Based Brushes

**Scheme 2:**
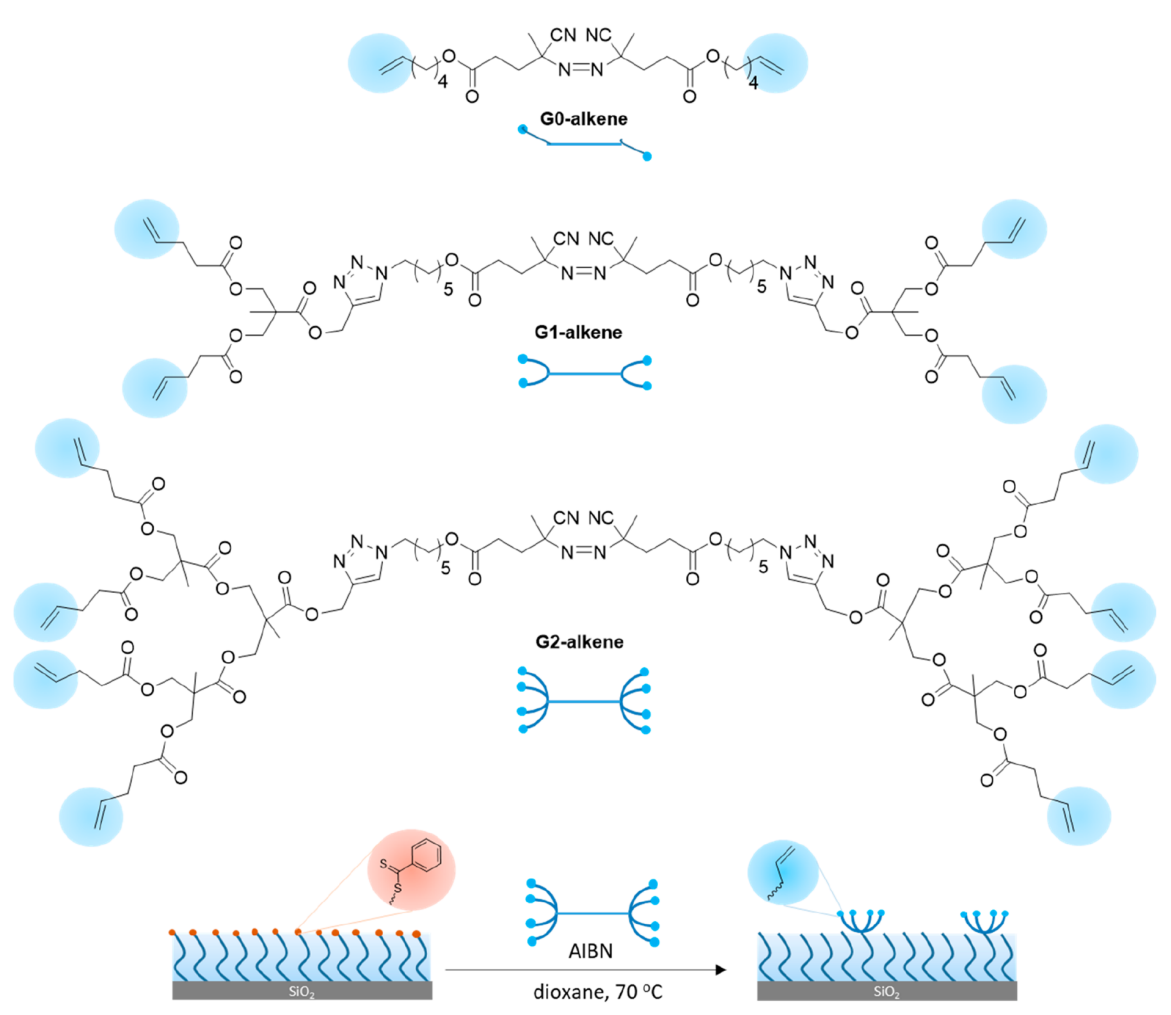
Structures of Dendritic Azo-Chain Terminators and Representative Surface Modification Using a G2-Alkene-Based Dendron-Grafted Brush

After successfully installing and functionalizing the “clickable” reactive groups at the interface, we envisioned that this approach could be utilized to install clusters of a functional group using dendritic azo-based chain end modifiers. To this end, we synthesized the azobis-G1-diene and azobis-G2-tetraene for utilization in post-polymerization modification (Scheme 2). Using the protocol established for the DEGMA polymer surfaces with azobis-ene (G0), surfaces were modified with azobis-G1-diene. A fixed amount of AIBN was added during the modification of brushes with azobis-G1-diene and azobis-G2-tetraene to reduce functional group crowding at the interface. After modification of the interface, surfaces were characterized using FTIR and XPS (Figure S17–20). Expected carbonyl (at 1724 cm^–1^) and alkene (at 1634 cm^–1^) stretching bands were observed in the FTIR spectra (Figure S27), and the appearance of N atoms (at ca. 400 eV) in XPS spectra indicated successful surface modification.

#### Functionalization with Mannose-SH via Thiol–Ene “Click” Chemistry

After obtaining the alkene-grafted dendron-modified surfaces, conjugation of D-Mannose-SH via the radical thiol–ene reaction was undertaken. After the modification, all surfaces were characterized with XPS spectroscopy to confirm the addition of the sugar units. After attachment of sugar to the G0-alkene interface, in the high-resolution XPS, the C 1s peak could be deconvoluted into four Gaussians with the expected relative areas for three carbon atoms at 288.8, 286.4, and 285.0 eV for C=O, C–O–C, and C–C, respectively, along with the anomeric carbon O–C–O peak at 287.8 eV for the mannose moiety (Figure S21). A similar analysis for all mannose-modified surfaces suggested successful conjugation of the mannose groups onto these surfaces (Figures S22–S24).

#### Immobilization of Concanavalin A on Dendron Functionalized Polymer Brushes

**Figure 8:**
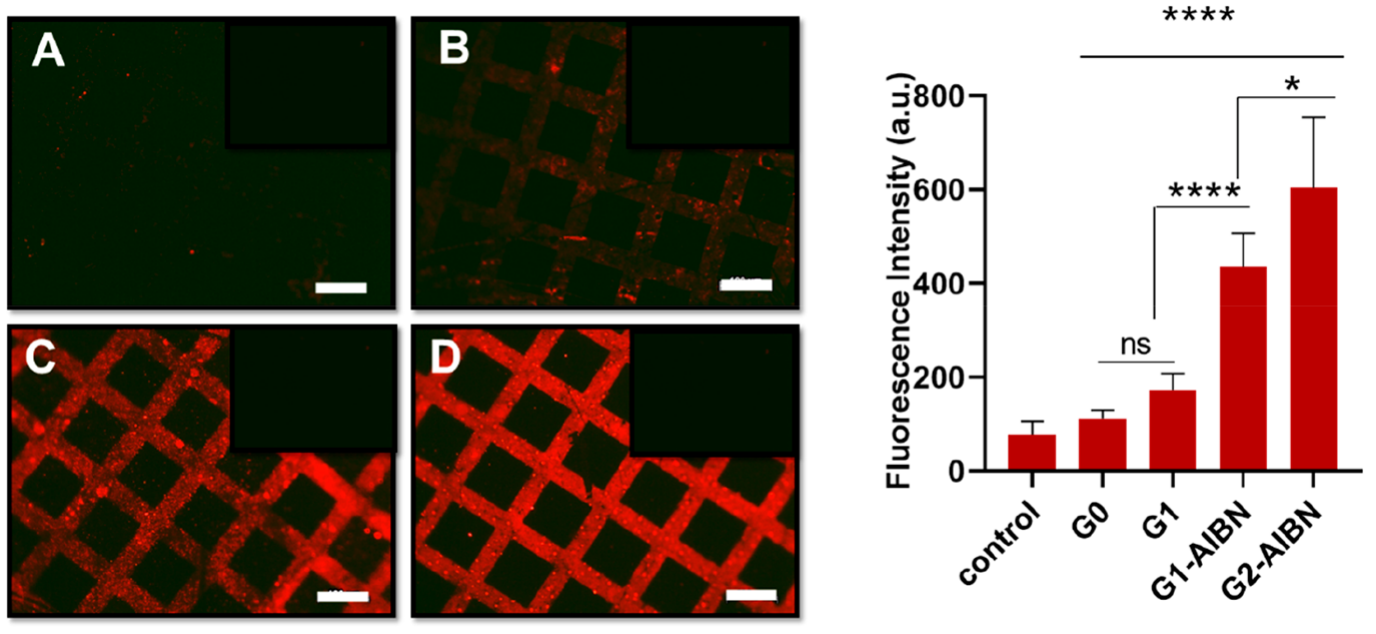
Treatment of mannose-terminated DEGMA polymer brushes with ConA. Fluorescence image of a micropatterned (A) DEGMA@G0ene-mannose polymer brush, (B) DEGMA@G1diene-mannose polymer brush, (C) DEGMA@G1diene/AIBN-mannose polymer brush, and (D) DEGMA@G2tetraene/AIBN-mannose polymer brush after immobilization of Texas Red conjugated ConA (scale bar is 100 μm). Statistical significance *P ≤ 0.05; **P ≤ 0.01; ***P ≤ 0.001; ****P ≤ 0.0001; and ns: *P* > 0.05.

Mannose-functionalized surfaces were treated with the Texas Red conjugated ConA protein solution in PBS buffer (pH 7.4, Mn^2+^ and Ca^2+^ containing buffer), and surfaces were characterized with fluorescence microscopy after rinsing with copious amounts of water to remove any unbound protein. While there was no significant attachment of ConA on surfaces devoid of the mannose group, there was almost no protein immobilization on the interface where single mannose units were installed. This is in contrast to the successful immobilization of streptavidin as demonstrated earlier, but not surprising considering the relatively poor binding of the mannose-ConA couple as compared to the biotin–streptavidin couple (*K*_d_ = 2.89 × 10^–6^ M and 1 × 10^–14^ M, respectively).^50,51^ Although from the fluorescence microscopy analysis, it was evident that, for the surface obtained using mannose modification of the G1-alkene, a slightly higher amount of ConA binding took place and it did not appear to be effective. To understand if the crowding of functional groups leads to this lack of enhanced protein binding, we decided to dilute the mannose clusters by adding AIBN during the attachment of the alkene units. To our surprise, the diluted mannose-conjugated dendritic surfaces performed significantly better (Figure 8). There was a considerably higher amount of protein immobilization on the G1-ene/AIBN modified surface compared to the surface modified with G1-ene alone. These experiments demonstrate that the presentation of the protein binding ligands in a dendritic fashion, without overcrowding, can enable significant improvement in ligand-directed protein immobilization and detection.

**Figure 9:**
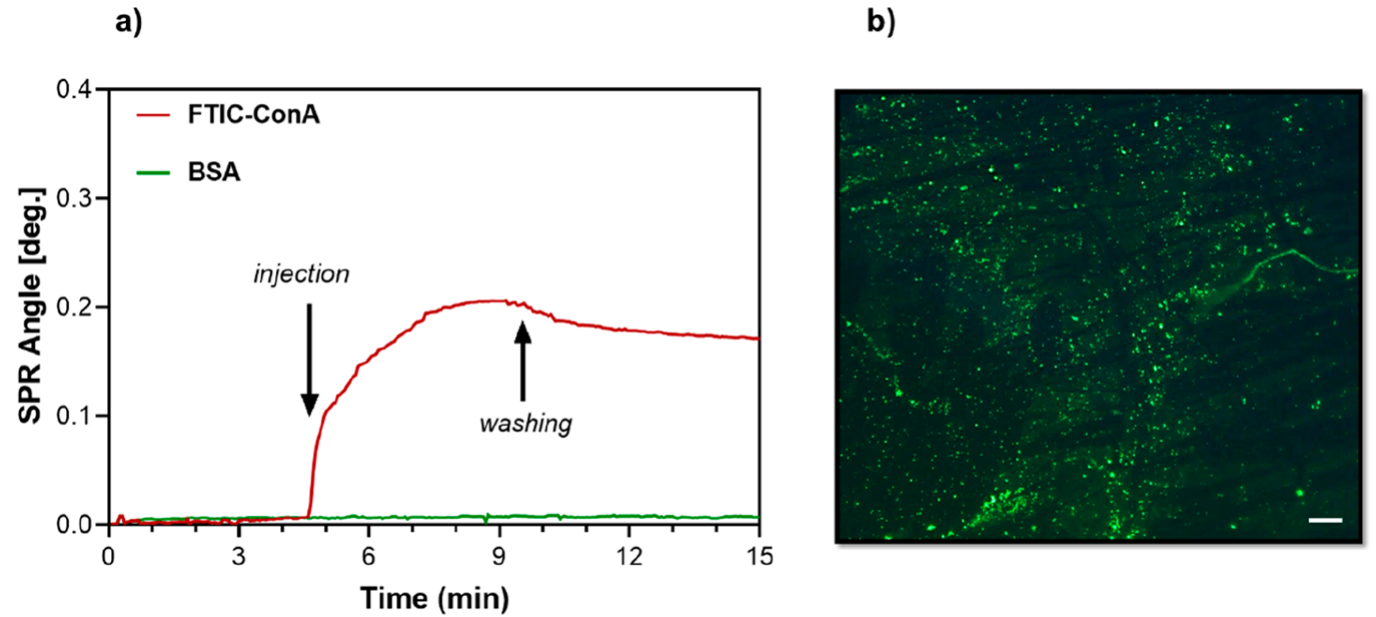
(a) Surface plasmon resonance (SPR) binding analysis indicating the interaction between mannose-bearing G2 polymer brushes with the two different proteins (FITC-ConA and BSA) and (b) fluorescence microscope image of SPR sensor chip after binding of FITC-ConA. (Scale bar is 100 μm).

Additionally, the specific ligand-directed attachment of the protein ConA was also probed using surface plasmon resonance (SPR) analysis. It is well-known that the carbohydrate recognition site of ConA is specific for mannose and glucose. Also, no interaction with other proteins such as BSA is anticipated due to the underlying PEG-based matrix. Hence, we compared the difference in the interaction between ConA and BSA proteins to a mannose-decorated SPR chip. A FITC-ConA aqueous buffer solution (20 mM HEPES, 1.0 mM MnCI_2_, 1.0 mM CaCI_2_, 0.15 M NaCl, adjusted to pH 7.4) was injected three times with a continuous flow over the polymer brush-coated SPR chip. The same protocol was used to access the interaction of the mannose-conjugated brush-coated SPR chip surface with BSA. As seen in Figure 9a, while FITC-ConA binds to the mannose-conjugated G2-based polymer brushes on the chip surface, BSA does not show any binding to the mannose-modified surface. This observation is in accordance with earlier observations for similar SPR studies in the literature.^52^ Also, a green fluorescence was observed upon inspection of the FITC-ConA treated mannose-coated SPR chip using fluorescence microscopy (Figure 9b).

## Conclusion

Work reported here discloses a versatile approach for obtaining a variety of functionalizable polymer brush interfaces that can be readily conjugated with various small molecules and ligands, using different “click” reactions. Importantly, the approach preserves the underlying polymer brush matrix while only tailoring the surface, thus affording a library of surfaces that only vary in their surface functionalities. The radical exchange reaction with appropriately functionalized azo-containing reactive molecules is employed to install azide, maleimide, and terminal-alkene groups on the brush surface. While the azide group enables functionalization using the Cu-catalyzed and metal-free azide–alkyne cycloadditions, the maleimide and terminal alkene groups allow conjugation through thiol-maleimide and radical thiol–ene chemistry. Successful attachment of fluorescent dye as well as biotin ligands is undertaken. The latter also allows ligand-based immobilization/detection of the FITC-labeled protein streptavidin. Additionally, we demonstrate that biotin can also be used to direct the immobilization of nanostructures such as streptavidin-coated quantum dots. The versatility of this approach is shown finally through the immobilization of dendritic functional group motifs at the interface. Using the azo-exchange reaction, alkene-terminated dendrons of two different generations are conjugated at the interface. Functionalization of these with mannose-thiol is undertaken to show the enhanced effectiveness of dendritic interfaces toward target protein recognition. We envision that the facile and versatile approach outlined here will be of interest in engineering functional interfaces for various biomedical applications.

## Experimental Section

### Materials and Instrumentation

All chemicals were used as received unless specified. 4-Cyano-4-(phenylcarbonothioylthio)pentanoic acid and dimethylamino pyridine (DMAP) were purchased from Sigma-Aldrich. 3-Aminopropyltriethoxysilane was obtained from Across Organics. Di(ethylene glycol) methyl ether methacrylate (DEGMA) was purchased from Sigma-Aldrich and filtered over neutral aluminum oxide before use. Azobisisobutyronitrile (AIBN, Sigma-Aldrich) was recrystallized from methanol and dried under a vacuum. 4,4′-Azobis(4-cyanovaleric acid) (V-501) was purchased from Fluka. For dendron synthesis, 2,2-bis(hydroxymethyl)propionic acid (Bis-MPA), 2,2-dimethoxypropane, and 4-pentenoic anhydride were obtained from Sigma-Aldrich. 1-(3-(Dimethylamino)propyl)-3-ethylcarbodiimide hydrochloride (EDCI) and *N*,*N*′-dicyclohexylcarbodiimide (DCC) were purchased from Alfa-Aesar and from Sigma-Aldrich, respectively. Biotinylated hexa(ethylene glycol)-undecanethiol (HS(CH_2_)_11_(OCH_2_CH_2_)_6_NH-Biotin, Biotin-SH) was purchased from Nanoscience Instrument (Phoenix, AZ). Qdot 605 streptavidin conjugate was obtained from Invitrogen molecular probes. DBCO-PEG_4_-carboxyrhodamine and dibenzocyclooctyne-PEG_4_-biotin were obtained from Click Chemistry Tools. Concanavalin A (Texas Red Conjugate) was purchased from Thermo Fisher. Surface attachable RAFT agent,^47^ G1-OH,^53^ G2-OH,^53^ 6-azido-1-hexanol,^54^ azobis-azide,^55^ BODIPY-SH,^56^ BODIPY-alkyne,^57^ azobis-pMAL,^46^ and mannose-SH^58,59^ were synthesized according to literature procedures. Dichloromethane (DCM, CH_2_Cl_2_), ethanol, chloroform, dimethylformamide (DMF), *n*-hexane, toluene, and 1,4-dioxane were purchased from Merck. Anhydrous toluene, tetrahydrofuran (THF), and DCM were obtained from a SciMatCo purification system, and other solvents were dried over molecular sieves. XPS was carried out using a K-Alpha instrument (Thermo Scientific). FTIR spectroscopy analyses were performed on a Nicolet 380 (Thermo Fisher Scientific, Inc.) instrument equipped with a Harrick Scientific GATR accessory and a Ge crystal. AFM was done using a Nanosurf instrument. ^1^H NMR and ^13^C NMR spectra were obtained using a Varian 400 MHz or Bruker Avance Ultrashield 400 (400 MHz) spectrometer. Fluorescence microscopy was performed using a LD-A-Plan 10*×*/0.30 objective in a Zeiss Axio Observer inverted microscope (Zeiss Fluorescence Microscopy, Carl Zeiss Canada Ltd., Canada). The statistical processing of the data was performed by one-way ANOVA analysis using GraphPad Prism software. Details of synthesis and NMR spectra of dendrons, and XPS spectra can be found in the Supporting Information.

### Synthesis and Immobilization of Surface Attachable RAFT Agent

The surface RAFT agent was synthesized and initiator-modified substrates were prepared by following a previously reported procedure.^47^ First, silicon wafers (0.8 cm × 1.0 cm) were washed by sonicating for 5 min in acetone, ethanol, and deionized water, respectively, and dried under a stream of nitrogen. Subsequently, the silicon wafers were cleaned using a Novascan PSD Series UV/Digital Ozone System for 30 min. Next, cleaned silicon wafers were immersed in a 1 mM anhydrous toluene solution of the surface RAFT agent and kept in solution under a nitrogen atmosphere for 4 h at room temperature. Finally, the silicon wafers were washed by sonicating in dichloromethane (three times) and with deionized water (two times), and then they were dried under a stream of nitrogen. The RAFT-agent-coated wafers were kept under nitrogen for post-polymerization modification.

### SI-RAFT Polymerization of DEGMA

Di(ethylene glycol) methyl ether methacrylate (DEGMA, 1.5 g, 8 mmol) and AIBN (2.16 mg, 0.013 mmol) were dissolved in anhydrous DMF and purged with N_2_ for 20 min. In a separate vial, the RAFT-agent-coated wafers were purged with N_2_ for 10 min and wafers were treated with the solution of DEGMA/AIBN under nitrogen at 70 °C for 6 h. After that, wafers were washed with DMF and CH_2_Cl_2_ with the aid of sonication, and subsequently, they were dried under a flow of nitrogen. The DEGMA brushes were stored under nitrogen for post-polymerization modification.

### Post-polymerization Modification of the DEGMA-Brushes with Azobis-azide

Azobis-azide (Diazido-V501) was synthesized in two steps according to a previously reported procedure.^54,55^ For post-polymerization modification, azobis-azide (azobis-N_3_) was dissolved in 1,4-dioxane and purged with N_2._ In a separate vial, DEGMA brushes were purged with N_2_, and then brushes were treated with the solution of azobis-azide under nitrogen at 70 °C for 24 h. Azide-containing brushes were washed with 1,4-dioxane and dichloromethane (CH_2_Cl_2_) with the aid of sonication, and finally, azide-containing brushes were dried under a flow of nitrogen.

### Post-polymerization Modification of Azide-Terminated DEGMA-Brushes with BODIPY-alkyne

BODIPY-alkyne was synthesized by following a previously reported procedure.^57^ CuSO_4_ (0.056 mg) was dissolved in distilled water (6 μL). After BODIPY was dissolved in methanol (1.5 mL), CuSO_4_·5H_2_O solution was added to the resolution of BODIPY-alkyne. Sodium ascorbate (0.39 mg) was dissolved in distilled water (1 mL). Subsequently, the solution of sodium ascorbate was added to the BODIPY alkyne solution. Then, azide-terminated polymer brushes were treated with the solution of BODIPY-alkyne at room temperature overnight. Finally, to get rid of unreacted BODIPY-alkyne, the silicon wafers were washed with methanol, dichloromethane, and deionized water, respectively, and dried under a nitrogen flow.

### Post-polymerization Modification of Azide-Terminated DEGMA-Brushes with DIBO-Biotin and Streptavidin-Coated QDots

DIBO-biotin (0.1 mg) was dissolved in methanol (500 μL), and an azide-terminated brush was treated with the solution of DIBO-biotin at room temperature overnight. After that, the surface was washed with methanol and dichloromethane to remove unreacted DIBO-biotin and then dried under a nitrogen flow. Next, a solution of streptavidin-coated CdSe Qdots (10 μL, 1 μM) dissolved in 10 μL of deionized water was placed onto the biotinylated surface and incubated for 6 h. Later, the surface was rinsed with deionized water (three times) to get rid of physisorbed nanoparticles.

### Typical Radical Cross-Coupling End-Group Modification of DEGMA-Brushes with Azobis-*p*-maleimide

Azobis-*p*-maleimide (0.18 mg, 0.025 mmol) was dissolved in a mixture of DMF/1,4-dioxane (1 mL, v/v) and purged with N_2_ for 20 min. In a separate vial, the DEGMA polymer surface was purged with N_2_ for 10 min, and the brush was treated with the solution of azobis-*p*-maleimide. Afterward, the surface was washed with DMF, dichloromethane, and deionized water, respectively, and dried under a nitrogen flow.

### Activation of Maleimide Functional Groups via Retro-Diels–Alder Reaction

The protected-maleimide-terminated polymer brush was placed into a vacuum oven and heated at 110 °C for 4 h. The maleimide-containing surface was washed with ethanol and dichloromethane by sonication and dried under a stream of nitrogen.

### Post-polymerization Modification of Maleimide-Containing DEGMA-Brushes with BODIPY-SH via Michael Addition

The maleimide-containing polymer brush surface was immersed in a solution of BODIPY-thiol in THF (1 mg/mL, 2.3 mM) and left overnight. Afterward, the surface was washed with THF to remove unreacted BODIPY-thiol and then dried under a stream of nitrogen.

### Post-polymerization Modification of Maleimide-Containing DEGMA-Brushes with Streptavidin-Coated QDots

Biotinylated hexa(ethylene glycol) undecanethiol (Biotin-SH) was dissolved in methanol (0.5 mg/mL), and the maleimide containing DEGMA polymer surface was treated with a solution of Biotin-SH for 24 h. After that, the surface was washed with methanol and dichloromethane and dried under a stream of nitrogen. Subsequently, a solution of Qdot (10 μL, 1 μM) dissolved in 10 μL of water was placed onto the biotinylated surface and incubated for 4 h. After this, the surface was rinsed with deionized water several times and dried under a stream of nitrogen.

### Post-polymerization Modification of DEGMA-Brushes with Alkene Grafted Dendrons

DEGMA polymer brushes were modified with azobis-G0-ene, azobis-G1-diene, azobis-G1-diene/AIBN, and azobis-G2-tetraene/AIBN, separately. DEGMA polymer brushes were treated with solutions of azobis-G0-ene (22 mg, 0.05 mmol), azobis-G1-diene (30 mg, 0.025 mmol), azobis-G1-diene/AIBN (30 mg, 0.025 mmol/4.1 mg, 0.025 mmol (0.025 M AIBN) and azobis-G2-tetraene/AIBN (25 mg, 0.0125 mmol/2.05 mg, 0.0125 mmol) (0.0125 M AIBN) in dioxane (1 mL) under nitrogen at 70 °C for 24 h. After 24 h, surfaces were washed with DMF, CH_2_Cl_2_, and deionized water.

### Post-polymerization Modification of Alkene Grafted Dendrons with Mannose-SH

Mannose-SH was synthesized in three steps according to a previously described procedure.^58,59^ Mannose-SH (3 mg, 0.01 mmol) and DMPA (0.5 mg, 2 × 10^–3^ mmol) were dissolved in DMF (60 μL), and after this solution was dropped onto a double bond grafted dendron modified polymer surface, surfaces were exposed to UV light for 30 min. After that, surfaces were washed with DMF and CH_2_Cl_2_ with the aid of sonication. The same protocol was used to control the DEGMA-containing polymer surface.

### Immobilization of ConA onto Mannose Functionalized Brushes

Mannose functionalized polymer surfaces were treated with 50 μL ConA-Texas red conjugate solution in PBS buffer (0.5 mg/mL) for 6 h and washed with an excess amount of PBS buffer. For the control experiment, a double bond grafted G2 dendron functionalized polymer surface was used, and this surface was also treated with ConA-Texas red conjugate in the same manner.

### Preparation of Mannose Grafted Brushes on the SPR Chip Surface

The mannose-containing polymer brushes on the SPR chip were prepared using the same reaction pathway used to prepare polymer brushes on the silicon surface. First, a clean SiO_2_-coated gold SPR chip was immersed in 1 mM anhydrous toluene solution of the surface RAFT agent and kept in solution under nitrogen atmosphere for 4 h at room temperature. Then, DEGMA polymer brushes were obtained as a result of RAFT polymerization. End group modification of DEGMA brushes on SPR chip was carried out using azobis-G2-tetraene. Mannose-bearing G2 grafted polymer brushes were obtained using thiol–ene click chemistry in the presence of mannose-thiol.

### SPR Measurement

Surface plasmon resonance (SPR) was performed for mannose–ConA binding affinity constant assay on a SPR biosensor instrument BioNavis (MP-SPR Navi 210A VASA). G2 dendron grafted polymer brushes were prepared using a SiO_2_-coated gold SPR sensor chip. The mannose decorated SPR chip was obtained with the conjugation of mannose-thiol to the end groups of G2 polymer brushes. In the SPR biosensor, the mannose-containing gold SPR chip was exposed to HEPES buffer (20 mM) until a stable baseline was obtained. To assess the binding of ConA to mannose units on the chip, an FTIC-ConA solution (0.8 mg/mL) in an aqueous buffer solution (20 mM HEPES, 1.0 mM MnCI_2_, 1.0 mM CaCI_2_, 0.15 M NaCl, adjusted to pH 7.4) was injected three times (250 μL from 0.8 mg/mL FTIC-ConA solution) from over the sensor chip surface. The flow rate was set to 50 μL·min^–1^, and the contact and dissociation/washing times were set to 5 and 5 min, respectively. As a control experiment, bovine serum albumin (BSA) protein solution was injected over the mannose-bearing SPR chip.

## Supporting Information Available

Data related to the synthesis of dendrons, the growth of polymer brushes, and their ATR-FTIR and XPS spectra (PDF)
