# Supplementary material for: “Clickable” Polymer Brush Interfaces: Tailoring Monovalent to Multivalent Ligand Display for Protein Immobilization and Sensing
Source: Bioconjug Chem. 2022 Aug 22;33(9):1672–84. doi: 10.1021/acs.bioconjchem.2c00298 (PMC9501913; doi:10.1021/acs.bioconjchem.2c00298)
Supplement: Supplementary file 1 — bc2c00298_si_001.pdf [file bc2c00298_si_001.pdf]

Supporting Information

'Clickable' Polymer Brush Interfaces: Tailoring Monovalent to Multivalent Ligand Display for Protein Immobilization and Sensing

Aysun Degirmenci,[a] Gizem Yeter Bas,[a] Rana Sanyal,[a,b] and Amitav Sanyal *,[a,b]

[a] *Department of Chemistry, Bogazici University, Istanbul 34342, Turkey*

[b] *Center for Life Sciences and Technologies, Bogazici University, Istanbul 34342, Turkey*

Corresponding author: amitav.sanyal@boun.edu.tr

Table of Contents

Synthesis Part 4
NMR Spectra 8
XPS Spectra 13
FTIR Spectra 20
Calculation of Grafting Density 21

List of Figures

Figure S1. ^{1}H NMR spectrum of azobis-ene ($CDCl_3$).. 8
Figure S2. ^{13}C NMR spectrum of azobis-ene ($CDCl_3$). 9
Figure S3. ^{1}H NMR spectrum of G1-diene-alkyne ($CDCl_3$).. 9
Figure S4. ^{13}C NMR spectrum of G1-diene-alkyne ($CDCl_3$).. 10
Figure S5. ^{1}H NMR spectrum of Azobis-G1-diene ($CDCl_3$). 10
Figure S6. ^{13}C NMR spectrum of Azobis-G1-diene ($CDCl_3$). 11
Figure S7. ^{1}H NMR spectrum of G2-tetraene ($CDCl_3$). 11
Figure S8. ^{13}C NMR spectrum of G2-tetraene ($CDCl_3$).. 12
Figure S9. ^{1}H NMR spectrum of Azobis-G2-tetraene ($CDCl_3$). 12
Figure S10. ^{13}C NMR spectrum of Azobis-G2-tetraene ($CDCl_3$).. 13
Figure S11. High resolution N1s and O1s XPS spectra of DEGMA-containing brush. 13
Figure S12. a) 2D and b) 3D AFM images of patterned DEGMA polymer brush, and c) cross-sectional height analysis. 14
Figure S13. Time dependent growth of DEGMA polymer brush as a line graph...... 14
Figure S14. XPS survey and high resolution C1s and O1s spectra of azide functionalized DEGMA-containing brush. 15
Figure S15. XPS survey and high resolution C1s, O1s, and N1s spectra of protected maleimide functionalized DEGMA-containing brush. 15
Figure S16. XPS survey and high resolution C1s and O1s, spectra of maleimide functionalized DEGMA-containing brush. 16

Figure S17. XPS survey and high resolution C1s, N1s, and O1s, spectra of alkene (azobis-G0-ene) functionalized DEGMA-containing brush. 16
Figure S18. XPS survey and high resolution C1s, O1s, and N1s spectra of G1-diene functionalized DEGMA-containing brush. 17
Figure S19. XPS survey and high resolution C1s, O1s, and N1s spectra of G1-diene/AIBN functionalized DEGMA-containing brush. 17
Figure S20. XPS survey and high resolution C1s, O1s, and N1s spectra of G2-tetraene/AIBN functionalized DEGMA-containing brush... 18
Figure S21. XPS survey and high resolution C1s, O1s, N1s, and S2p, spectra of mannose-SH functionalized G0 (azobis-ene)-containing brush................................ 18
Figure S22. XPS survey and high resolution C1s, O1s, N1s, and S2p, spectra of mannose-SH functionalized G1-containing brush.. 19
Figure S23. XPS survey and high resolution C1s, O1s, N1s, and S2p, spectra of mannose-SH functionalized G1/AIBN-containing brush. 19
Figure S24. XPS survey and high resolution C1s, O1s, N1s, and S2p, spectra of mannose-SH functionalized G2/AIBN-containing brush. 20
Figure S25. FT-IR spectra of azide functionalized DEGMA containing brushes (green line), BODIPY-alkyne functionalized DEGMA containing brushes (orange line) and Rhodamine conjugated DBCO alkyne functionalized DEGMA containing brushes (black line). 20
Figure S26. FT-IR spectra of furan-protected maleimide functionalized DEGMA brushes (grey line), and maleimide functionalized DEGMA brushes after retro Diels-Alder reaction (dark blue line).. 21
Figure S27. FT-IR spectra of azobis-G0-ene functionalized DEGMA brushes (peach line), azobis-G1-diene functionalized DEGMA brushes (navy blue line), and azobis-G2-tetraene functionalized DEGMA brushes (green line).. 21

Synthesis Part

Synthesis of azobis-ene (azobis-G0-ene)

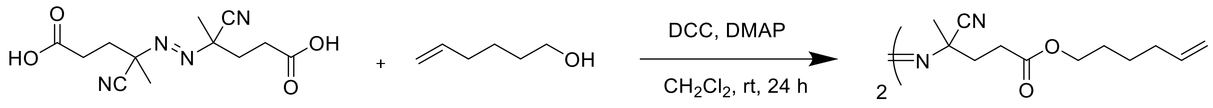

4,4′-Azobis(4-cyanovaleric acid) (V-501, 840 mg, 3 mmol), DMAP (146 mg, 1.2 mmol), and 5-hexen-1ol (660.4 mg, 6.6 mmol) were purged with N_2 for 15 min, and anhydrous dichloromethane (3 mL) was added under N_2. In a separate flask, DCC (1.26 g, 6.1 mmol) was dissolved in anhydrous dichloromethane (5 mL) under $N_{2,}$ and the solution was cooled at 0 ^oC. At 0 ^{o} C, the solution of DCC was added to the solution of V-501/DMAP/5-Hexen-1-ol. The reaction mixture was stirred at room temperature for 24 h. The reaction mixture was filtered to remove DCU, and then dichloromethane was evaporated. The crude product was re-dissolved in dichloromethane (50 mL) and extracted with $NaHSO_4$ solution (1 x 10 mL), Na_2CO_3 solution (1 x 10 mL) and brine (1 x 10 mL), respectively. The organic layer was dried over anhydrous Na_2SO_4, and concentrated under vacuum. The crude product was purified by column chromatography (EtOAc/Hexane, 4:6, v/v), (76 ± 4 % yield). ^{1}H NMR (400 MHz, $CDCl_3$) δ/ppm: 5.82-5.72 (m, 2H), 5.01-4.94 (m, 4H), 4.08 (br s, 4H), 2.49-2.25 (m, 8H), 2.08-2.01 (m, 4H), 1.7 (s, 3H),1.65-1.59 (m, 6H), 1.44-1.41 (m, 4H); ^{13}C NMR (100 MHz, $CDCl_3$) δ/ppm171.4, 138.2, 117.1, 114.9, 71.9, 65.0, 60.4, 33.2, 29.1, 27.9, 25.1, 23.9. FTIR (cm^{-1}): 3065, 2938,2857, 1730,1678, 1177.

Synthesis of G1-diene-alkyne

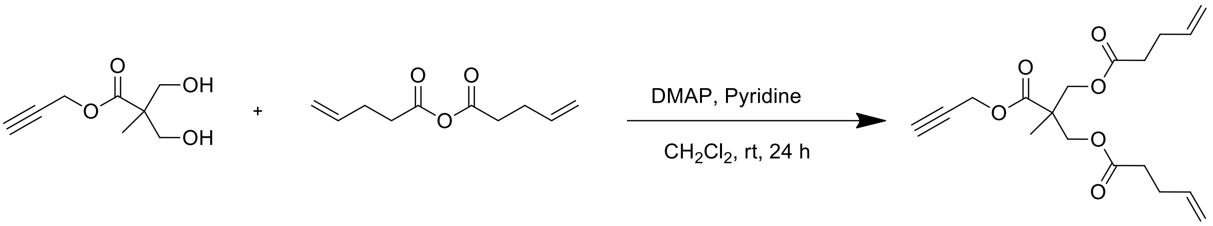

Prop-2-yn-1-yl-3-hydroxy-2-(hydroxymethyl)-2-methylpropanoate (G1-OH, 400 mg, 2.32 mmol), 4-pentenoic anhydride (2.1 g, 2 mL, 11.6 mmol), DMAP (113 mg, 0.93 mmol) and pyridine (917 mg, 0.94 mL, 11.6 mmol) were dissolved in dichloromethane (10 mL) and the reaction mixture was stirred at room temperature for 24 h. After that, deionized water (1 mL) was added and the mixture was stirred for 3 h. Thereafter, the mixture was poured into dichloromethane (50 mL) and extracted with $NaHSO_4$ solution (1 x 10 mL), Na_2CO_3 solution (1 x 10 mL), and brine (1 x 10 mL) respectively. The organic layer was dried over anhydrous Na_2SO_4. The organic layer was concentrated under vacuum. The crude product was purified by column chromatography (EtOAc/Hexane, 30:70, v/v). The pure product was obtained in 72 ± 2 % yield. ^{1}H NMR (400 MHz, $CDCl_3$) δ/ppm: 5.82-5.72 (m, 2H), 5.04-4.96 (m, 4H), 4.68 (s, 2H), 4.21 (s, 4H), 3,68 (s, 1H), 2.44-2.32 (m, 8H), 1.24 (s, 3H); ^{13}C NMR (100 MHz, $CDCl_3$) δ/ppm: 172.6, 172.4, 136.2, 115.6, 75.2, 65.2, 52.6, 46.4, 33.3, 28.7, 17.71.

Synthesis of Azobis-G1-diene

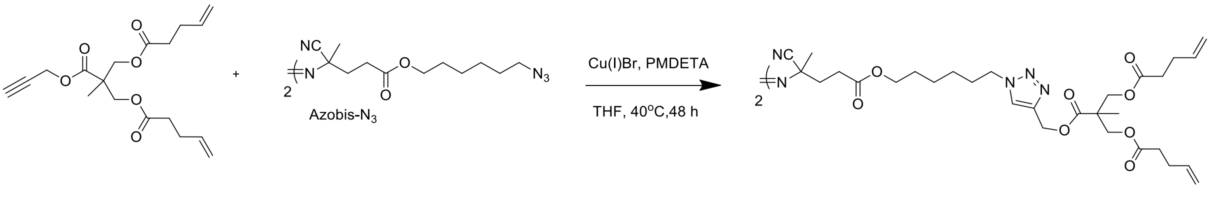

2-Methyl-2-((prop-2-yn-1-yloxy)carbonyl)propane-1,3-diyl bis(pent-4-enoate) (G1-diene, 250 mg, 0.74 mmol) and azobis-N_3 (180 mg, 0.34 mmol) were purged with N_2

and dissolved in anhydrous THF (2 mL) under N_2. In separate flask, Cu(I)Br (9 mg, 0.067 mmol) and PMDETA (14 μL, 0,067 mmol) were dissolved in anhydrous THF (4 mL) and purged with N_2. The solution of G1-diene and azobis-N_3 was added to the solution of Cu(I)Br and PMDETA under N_2. The reaction mixture was stirred at 40 ^oC for 24 h. After completion of the reaction, THF was removed under vacuo and the crude product was dissolved in dichloromethane (50 mL) and then extracted with brine (3 x 10 mL). The organic layer was dried over anhydrous Na_2SO_4. The crude product was purified by column chromatography (EtOAc/Hexane, 70:30, v/v) (yield: 82%). ^{1}H NMR (400 MHz, $CDCl_3$) δ/ppm: 7.57 (s, 2H), 5.83-5.73 (m, 4H), 5.25 (s, 4H), 5.05-4.98 (m, 8H), 4.34 (t, *J* = 7.1, 4H), 4.20 (q, *J1* =11.0, *J2* = 6.3, 8H), 4.12-4.04 (br s, 4H), 2.54-2.31 (m, 20H), 1.94-1.86 (m, 4H), 1.73 (s, 3H), 1.65-1.57 (m, 11H), 1.38 (br s, 8H), 1.2 (s, 6H). %. ^{13}C NMR (400 MHz, $CDCl_3$) δ/ppm: 172.7, 172.4, 171.3, 142.3, 136.4, 123.7, 117.5, 115.7, 71.8, 65.2, 64.8, 58.4, 50.3, 46.3, 33.3, 30.1, 29.1, 28.7, 28.4, 26.12, 25.3, 24.0, 23.7, 17.8. FT-IR (cm^{-1}): 3081, 2938, 1724, 1676, 1641, 1156, 1131, 1050 cm^{-1}.

Synthesis of G2-tetraene-alkyne

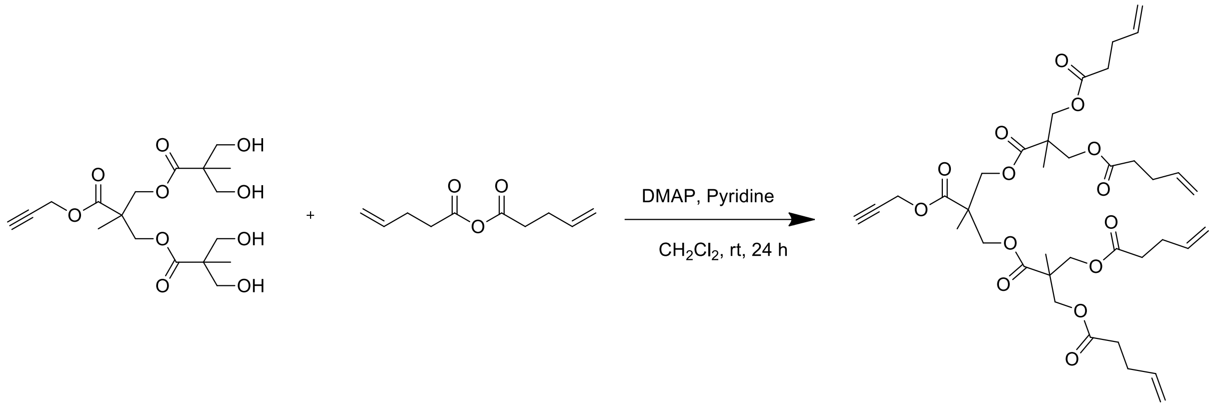

G2-OH-alkyne (450 mg, 1.11 mmol), 4-pentenoic anhydride (1.6 mL, 1.67 g, 8.91 mmol, DMAP (86 mg, 0.71 mmol), and pyridine (0.72 mL, 704 mg, 8.91 mmol) were dissolved in anhydrous dichloromethane (8 mL) and this mixture was stirred at room

temperature for 24 hours. After that, the reaction mixture was poured into dichloromethane (50 mL) and extracted with $NaHSO_4$, Na_2CO_3 and brine solution (1 x 10 mL), respectively. The organic layer was dried over anhydrous Na_2SO_4. The crude product was purified using column chromatography (EtOAc/ Hexane 30:70 v/v). G2-tetraene-alkyne was obtained with a 67 ± 5 % yield. ^{1}H NMR (400 MHz, $CDCl_3$) δ/ppm: 5.83-5.73 (m, 4H), 5.05-4.96 (m, 8H), 4.67 (s, 2H), 4.29-4.16 (m, 12H), 2.48-2.32 (m, 16H), 1.26 (s, 3H), 1.21 (s, 6H); ^{13}C NMR (100 MHz, $CDCl_3$) δ/ppm: 172.4, 172.0, 171.4, 136.4, 115.6, 75.6, 65.7, 65.2, 52.8, 46.7, 46.4, 33.3, 28.7, 17.9, 17.5.

Synthesis of Azobis-G2-tetraene

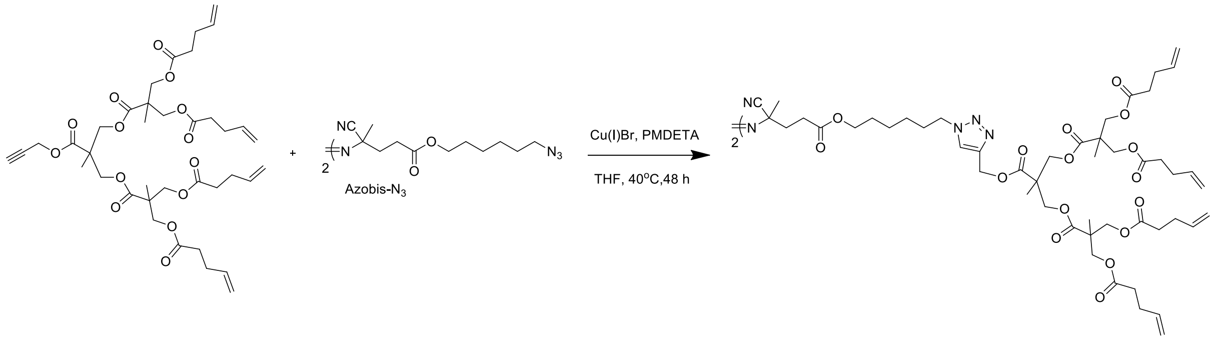

G2-tetraen-alkyne (150 mg, 0.22 mmol) and azobis-N_3 (52 mg, 0.098 mmol) were dissolved in anhydrous THF (4 mL) and purged with N_2 for 10 min. In a separate flask, Cu(I)Br (2.81 mg, 0.02 mmol) and PMDETA (4.2 μL, 3.47 mg, 0.02 mmol) were dissolved in anhydrous THF (4 mL) and purged with N_2 for 10 min. Finally, the solution of G2-tetraene-alkyne and azobis-N_3 was added to the solution of PMDETA and Cu(I)Br under nitrogen. The reaction mixture was stirred at 40 ^{o}C for 24 h. After that, the reaction mixture was poured into dichloromethane (50 mL) and extracted with $NaHSO_4$, Na_2CO_3 and brine solution (1 x 10 mL), respectively. The organic layer was dried over anhydrous Na_2SO_4. The crude product was purified using column chromatography (EtOAc/ Hexane 60:40 v/v). The pure product was obtained as a

colorless liquid in 76 ± 5 % yield. (400 MHz, $CDCl_3$) δ/ppm: 7.65 (s, 2H), 5.82-5.72 (m,8H), 5.23 (s, 4H), 5.04-4.96 (m, 16H), 4.35 (t, *J* = 7.0, 4H), 4.25-4.07 (m, 26H), 2.53-2.30 (m, 38H), 1.95-1.88 (m, 4H), 1.71 (s, 3H), 1.65 (s, 6H), 1.36 (s,10H), 1.21 (s, 6H), 1.17 (s, 12H). ^{13}C NMR (100 MHz, $CDCl_3$) δ/ppm: 172.5, 172.2, 172.0, 142.0, 136.4, 124.1, 115.7, 72.0, 65.6, 65.1, 64.9, 58.5, 50.3, 46.7, 46.3, 33.2, 30.2, 29.2, 29.1, 28.7, 28.3, 26.2, 25.4, 24.0, 23.7, 17.8, 17.5. FT-IR (cm^{-1}): 3081, 2938, 1724, 1676, 1641, 1156, 1131, 1050 cm^{-1}.

NMR Spectra

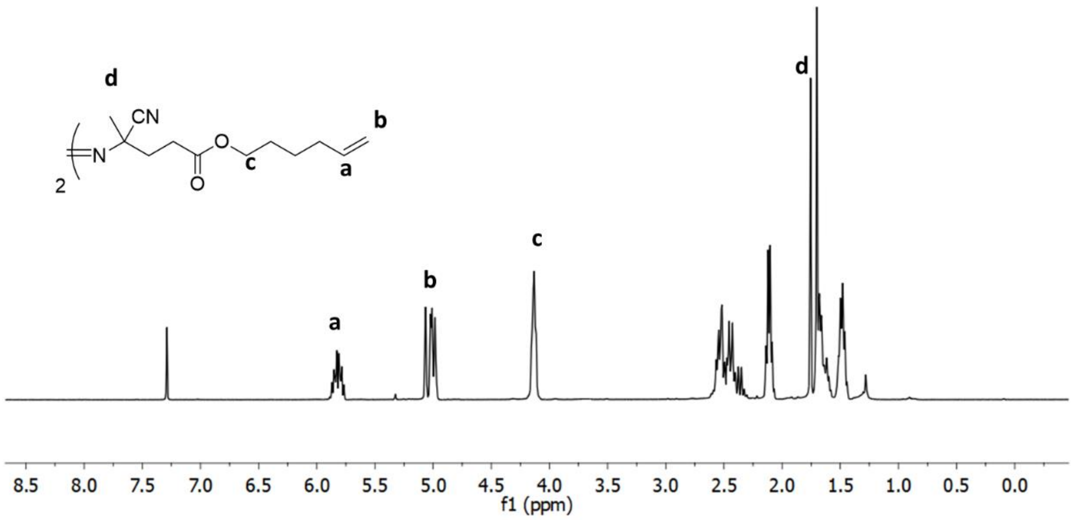

Figure S1. ^{1}H NMR spectrum of azobis-ene ($CDCl_3$).

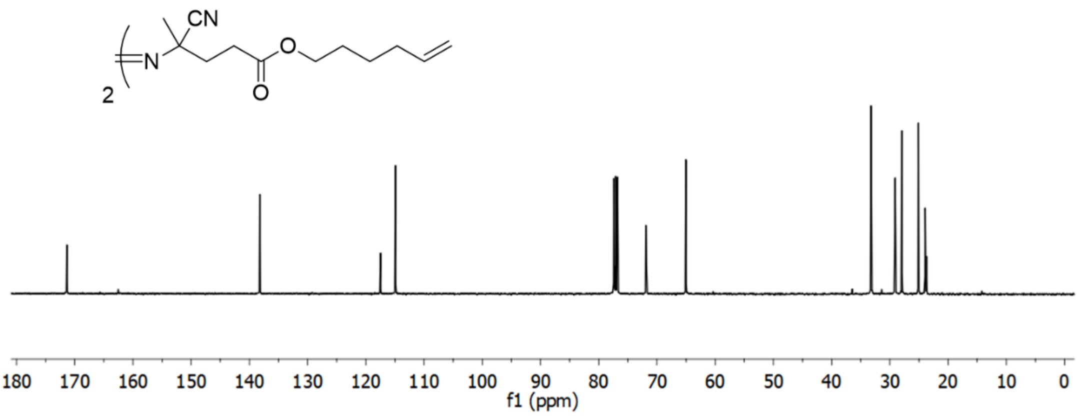

Figure S2. ^{13}C NMR spectrum of azobis-ene ($CDCl_3$).

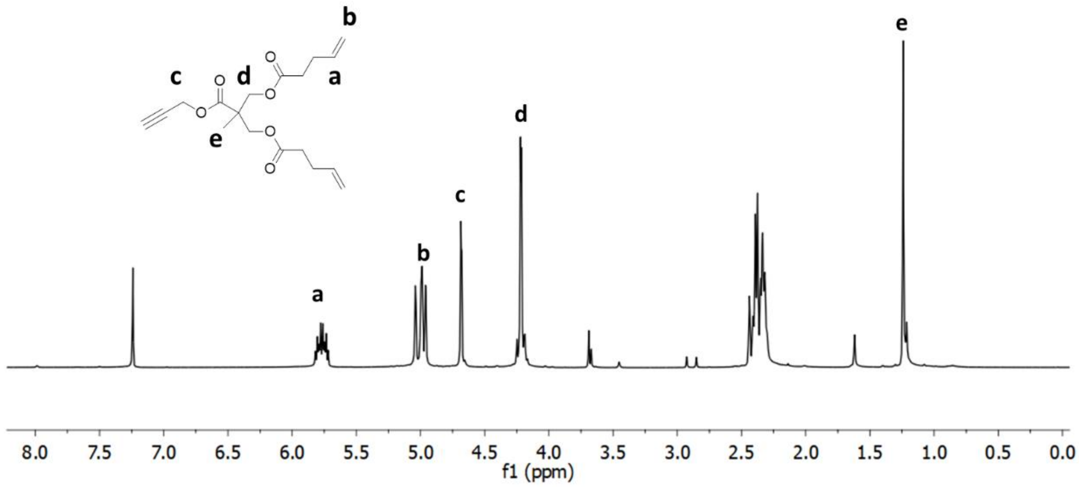

Figure S3. ^{1}H NMR spectrum of G1-diene-alkyne ($CDCl_3$).

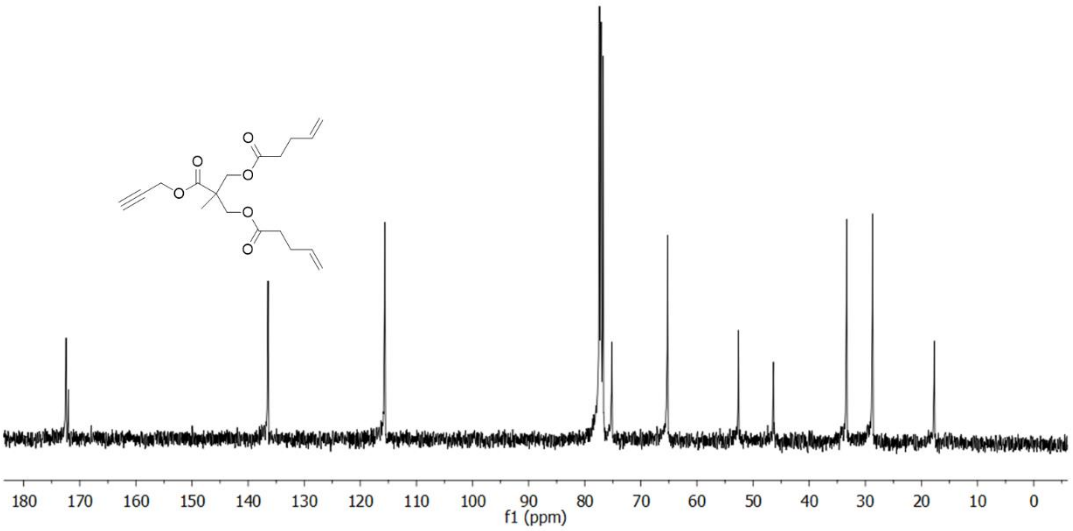

Figure S4. ^{13}C NMR spectrum of G1-diene-alkyne ($CDCl_3$).

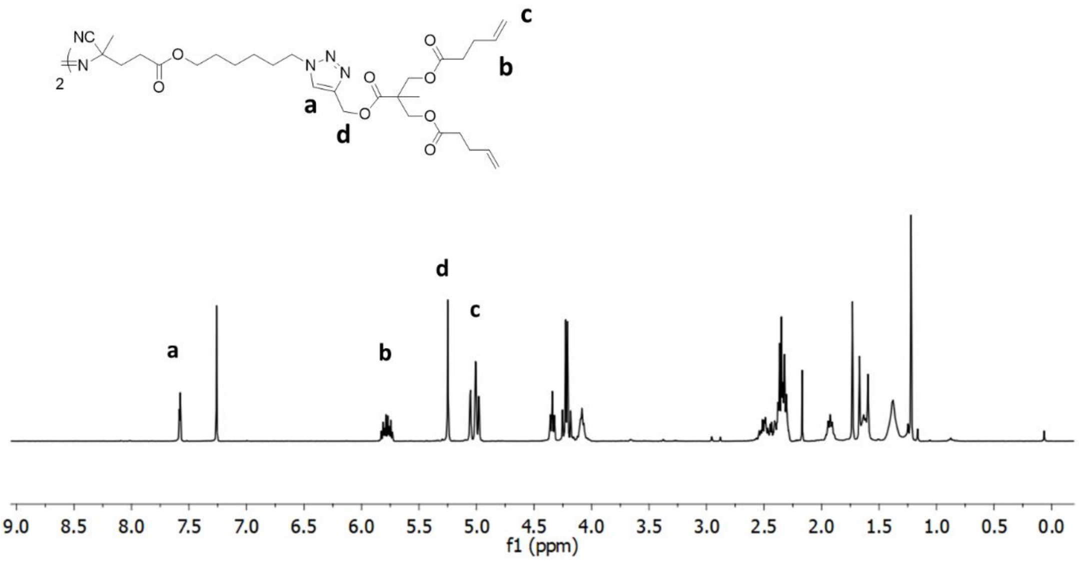

Figure S5. ^{1}H NMR spectrum of Azobis-G1-diene ($CDCl_3$).

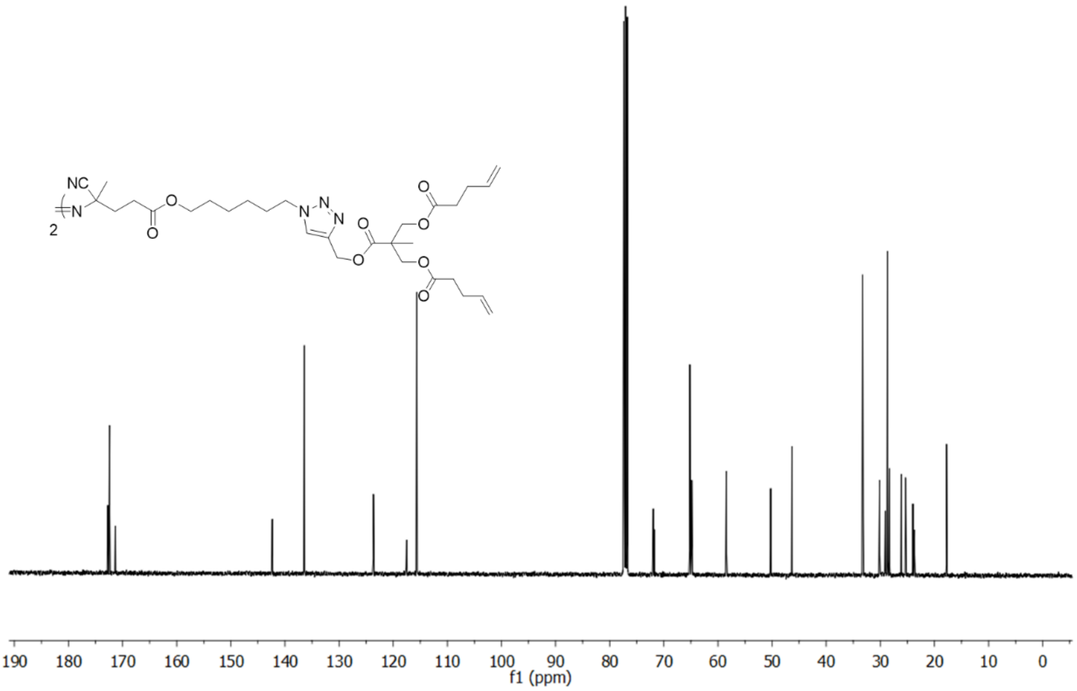

Figure S6. ^{13}C NMR spectrum of Azobis-G1-diene ($CDCl_3$).

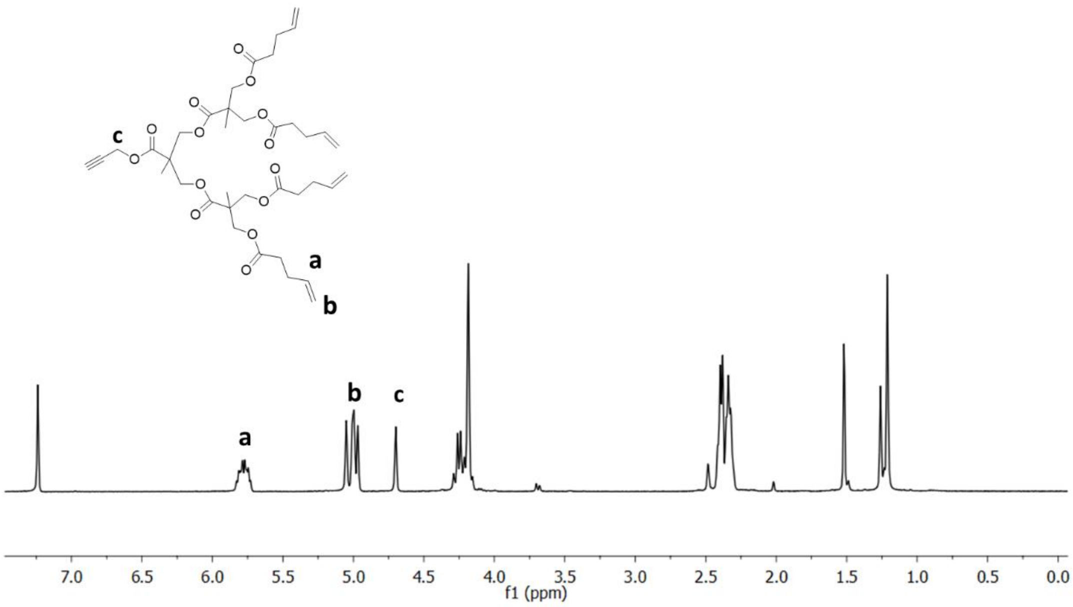

Figure S7. ^{1}H NMR spectrum of G2-tetraene ($CDCl_3$).

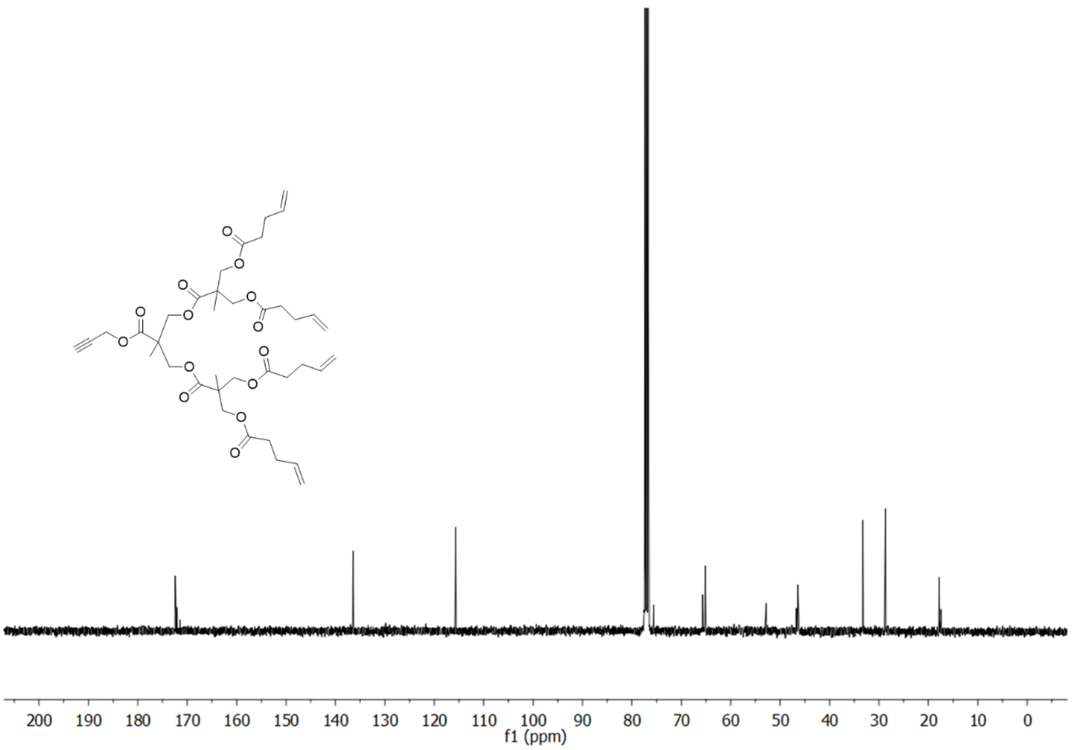

Figure S8. ^{13}C NMR spectrum of G2-tetraene ($CDCl_3$).

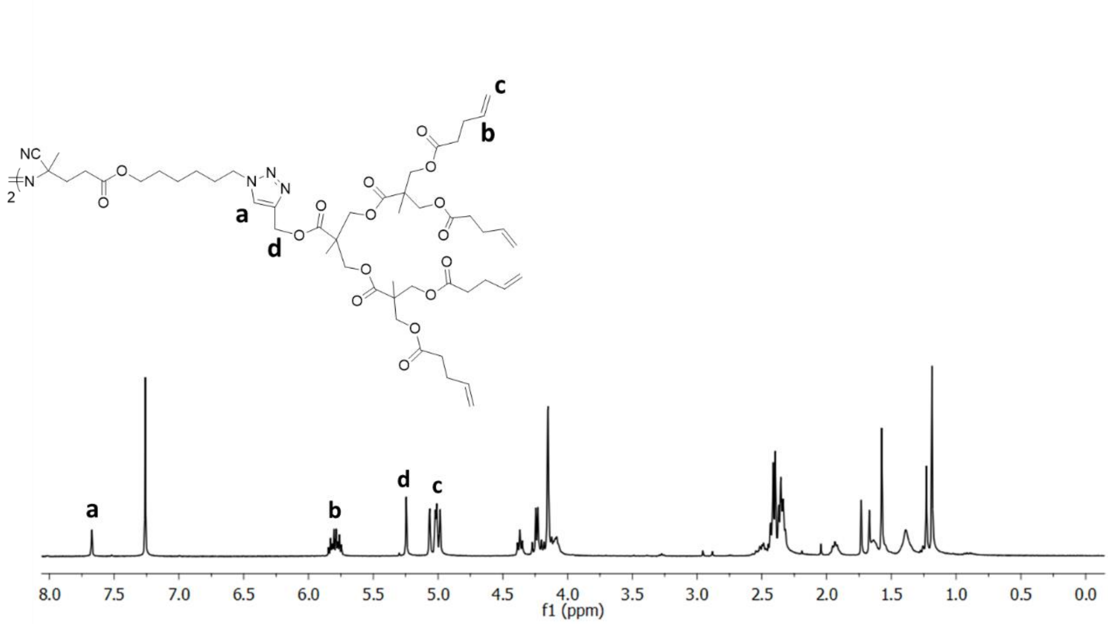

Figure S9. ^{1}H NMR spectrum of Azobis-G2-tetraene ($CDCl_3$).

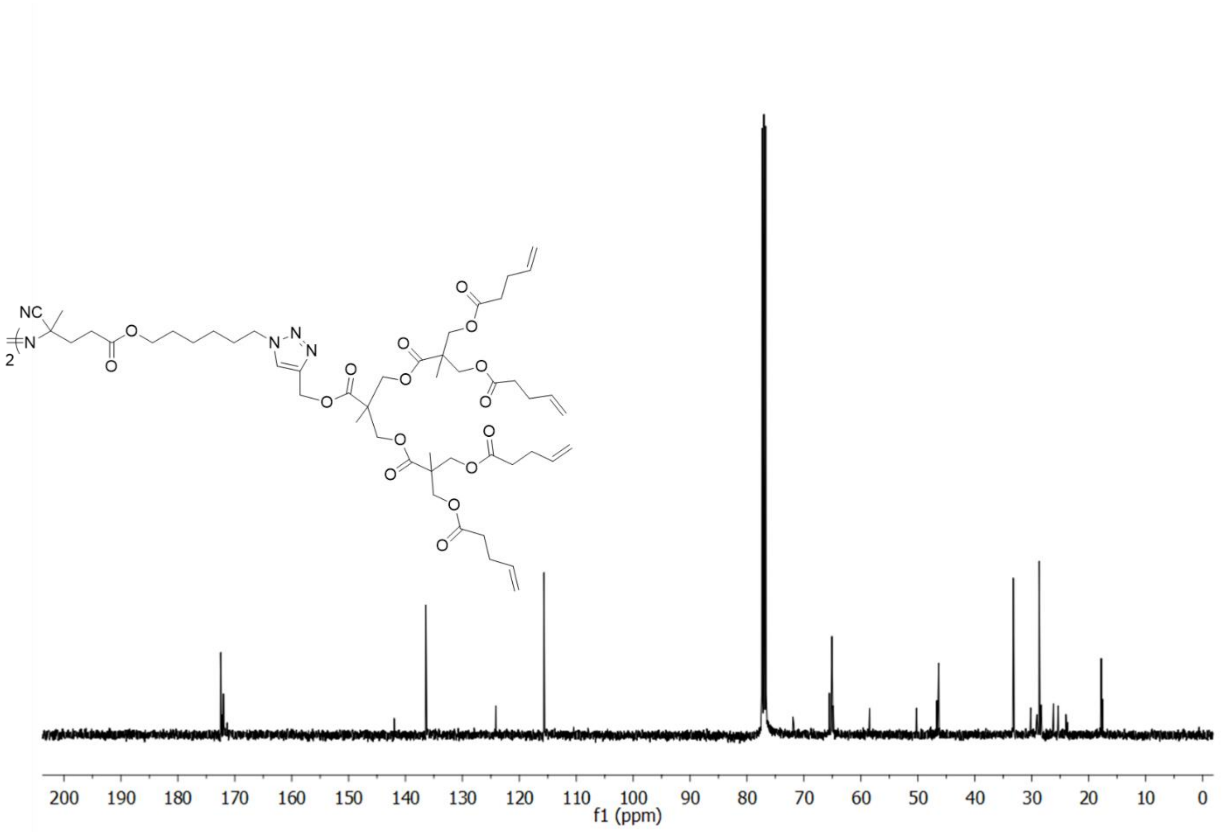

Figure S10. ^{13}C NMR spectrum of Azobis-G2-tetraene ($CDCl_3$).

XPS Spectra

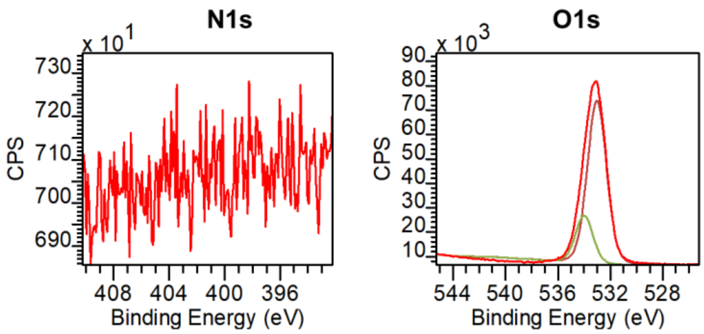

Figure S11. High resolution N1s and O1s XPS spectra of DEGMA-containing brush.

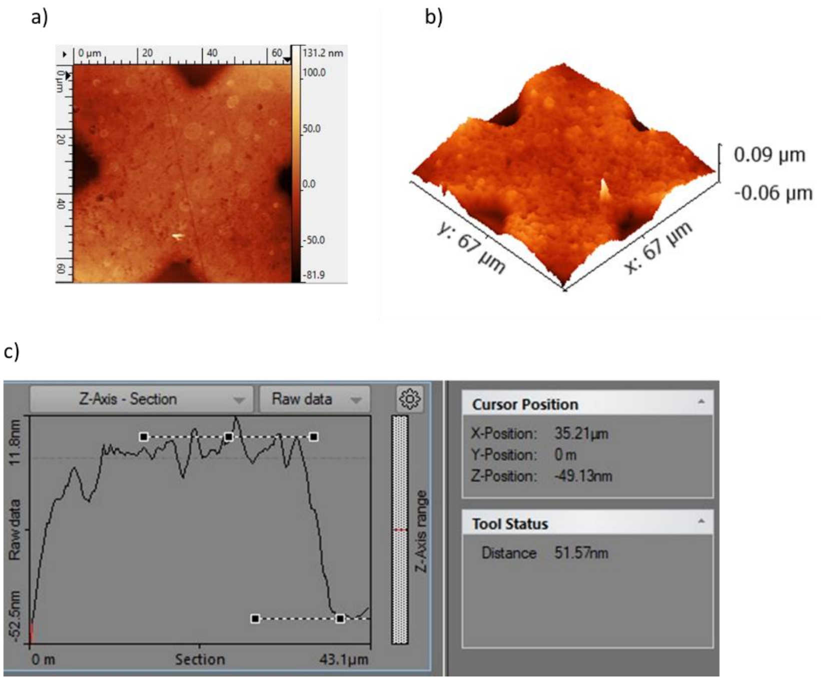

Figure S12. a) 2D and b) 3D AFM images of patterned DEGMA polymer brush, and c) cross-sectional height analysis.

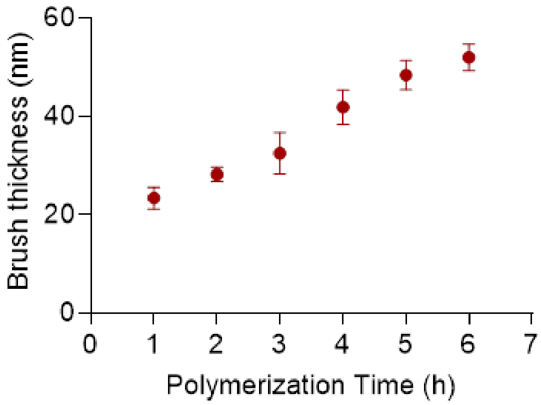

Figure S13. Time dependent growth of DEGMA polymer brush as a line graph.

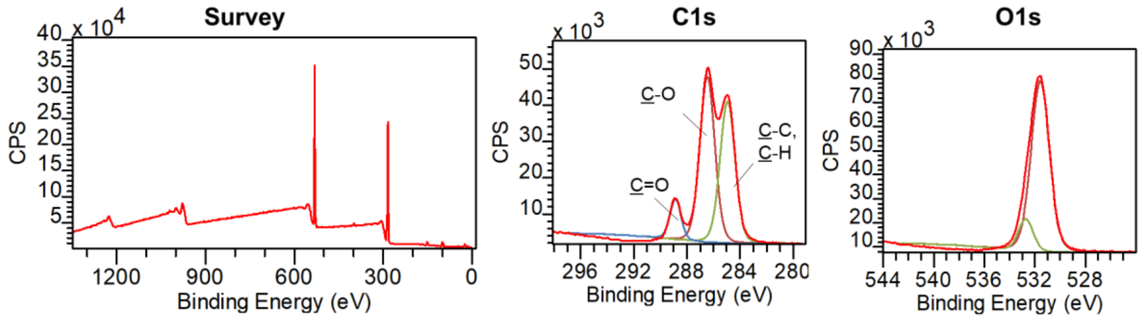

Figure S14. XPS survey and high resolution C1s and O1s spectra of azide functionalized DEGMA-containing brush.

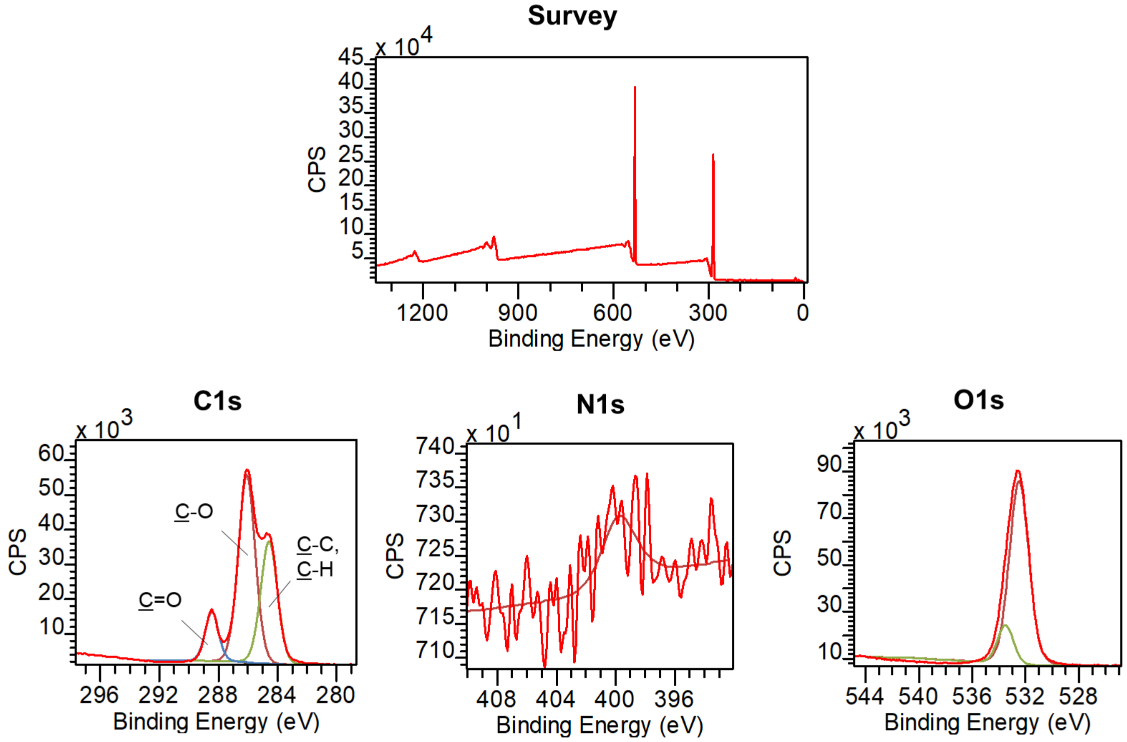

Figure S15. XPS survey and high resolution C1s, O1s, and N1s spectra of protected maleimide functionalized DEGMA-containing brush.

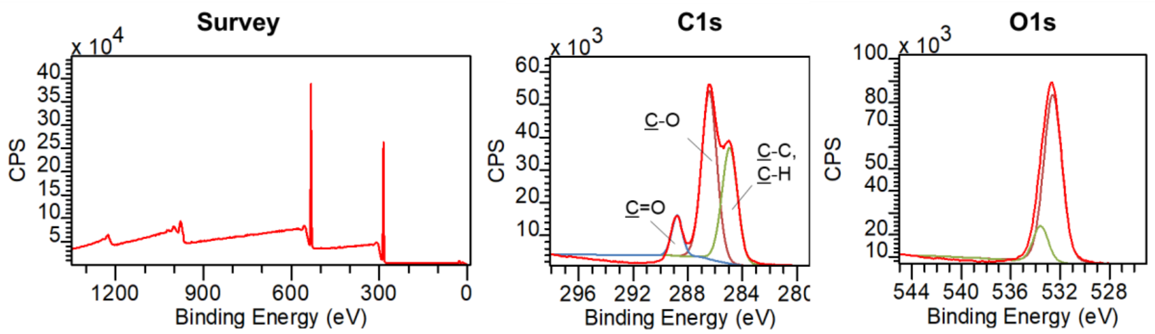

Figure S16. XPS survey and high resolution C1s and O1s, spectra of maleimide functionalized DEGMA-containing brush.

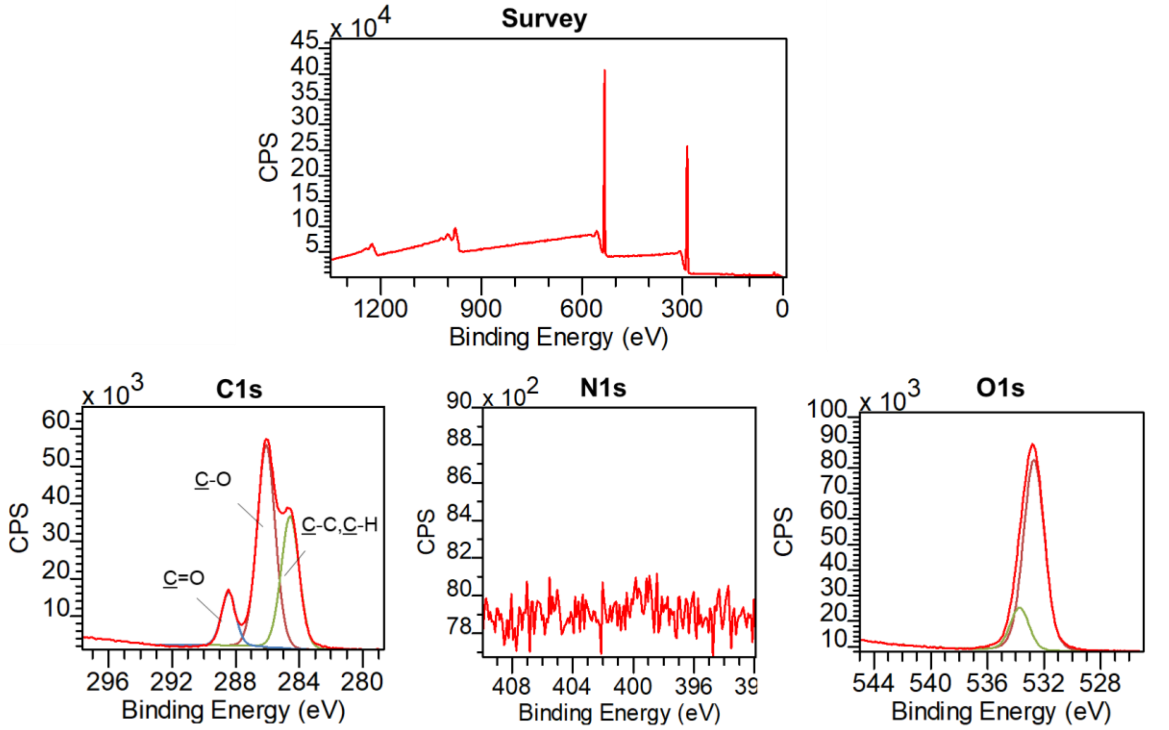

Figure S17. XPS survey and high resolution C1s, N1s, and O1s, spectra of alkene (azobis-G0-ene) functionalized DEGMA-containing brush.

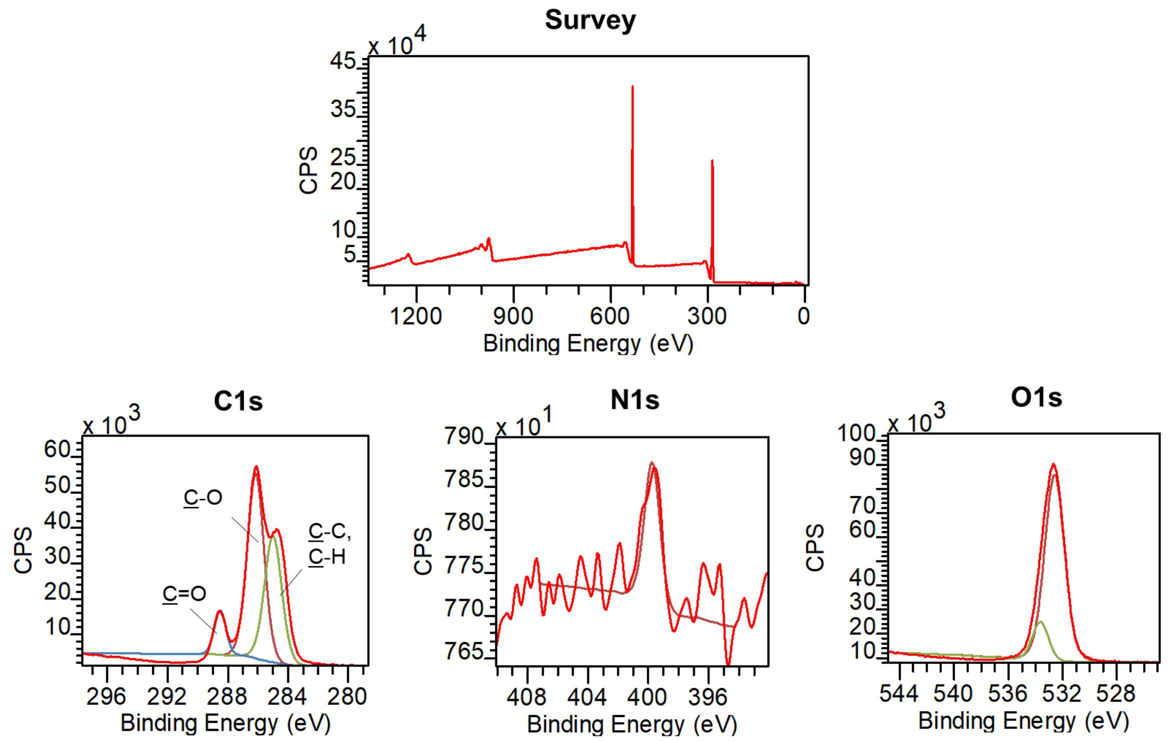

Figure S18. XPS survey and high resolution C1s, O1s, and N1s spectra of G1-diene functionalized DEGMA-containing brush.

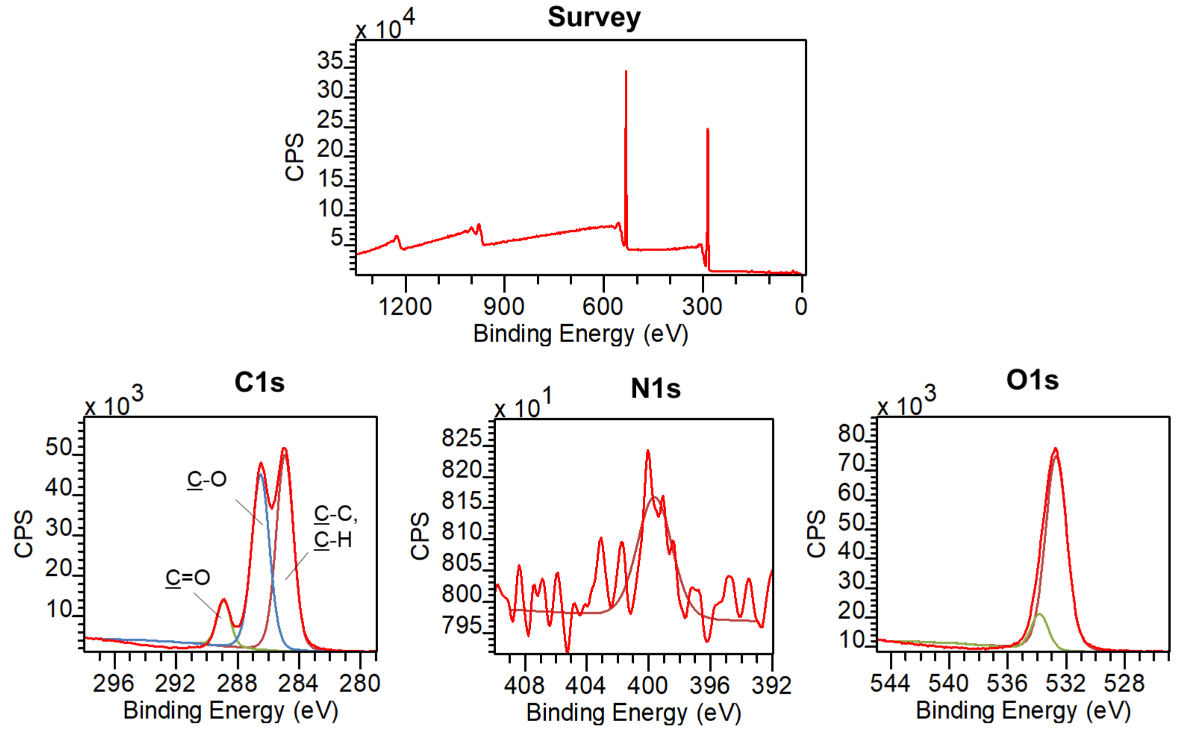

Figure S19. XPS survey and high resolution C1s, O1s, and N1s spectra of G1-diene/AIBN functionalized DEGMA-containing brush.

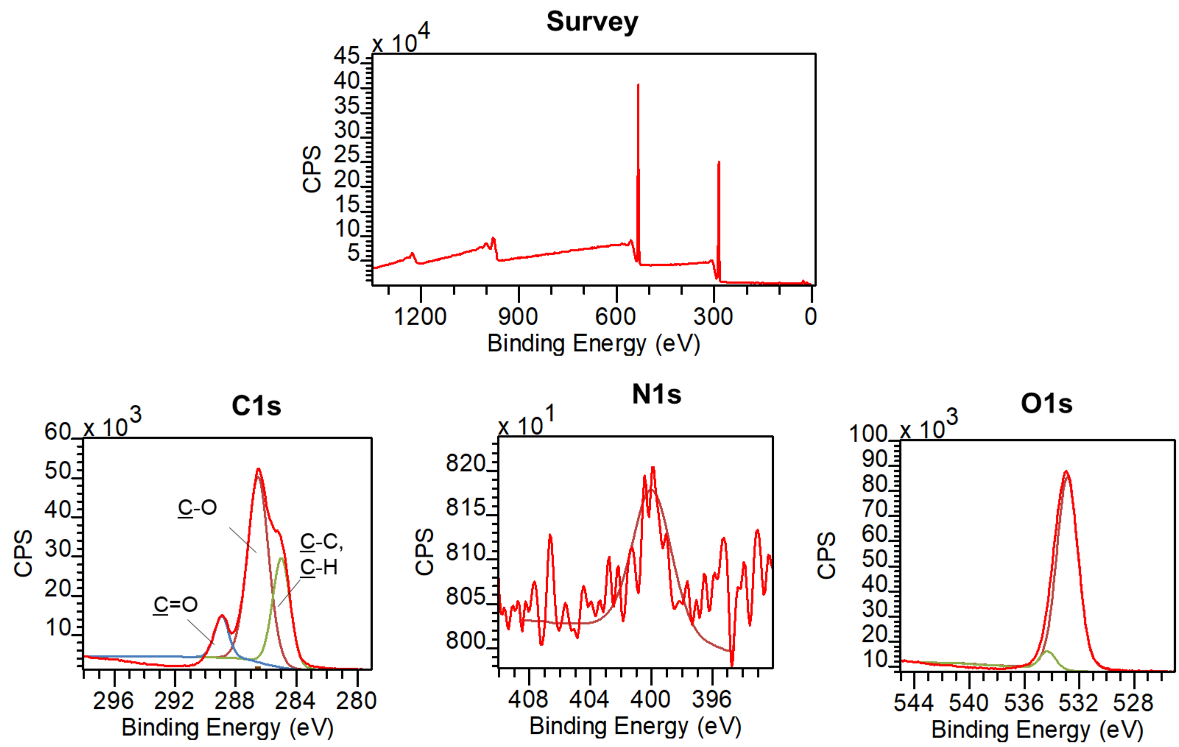

Figure S20. XPS survey and high resolution C1s, O1s, and N1s spectra of G2-tetraene/AIBN functionalized DEGMA-containing brush.

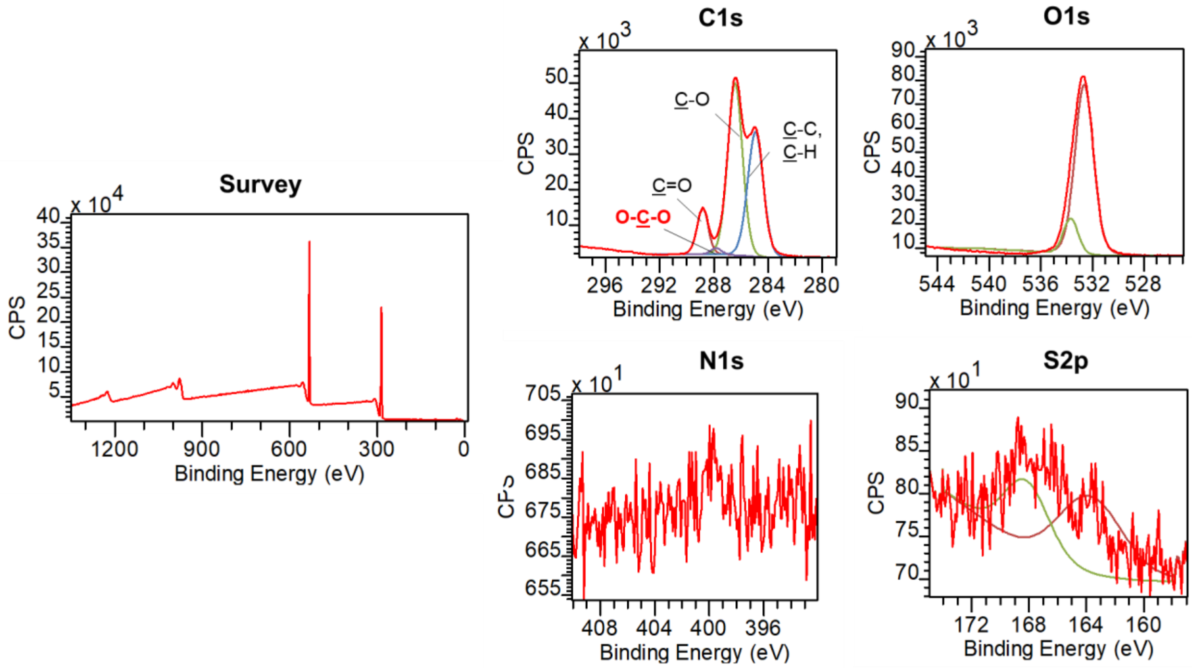

Figure S21. XPS survey and high resolution C1s, O1s, N1s, and S2p, spectra of mannose-SH functionalized G0 (azobis-ene)-containing brush.

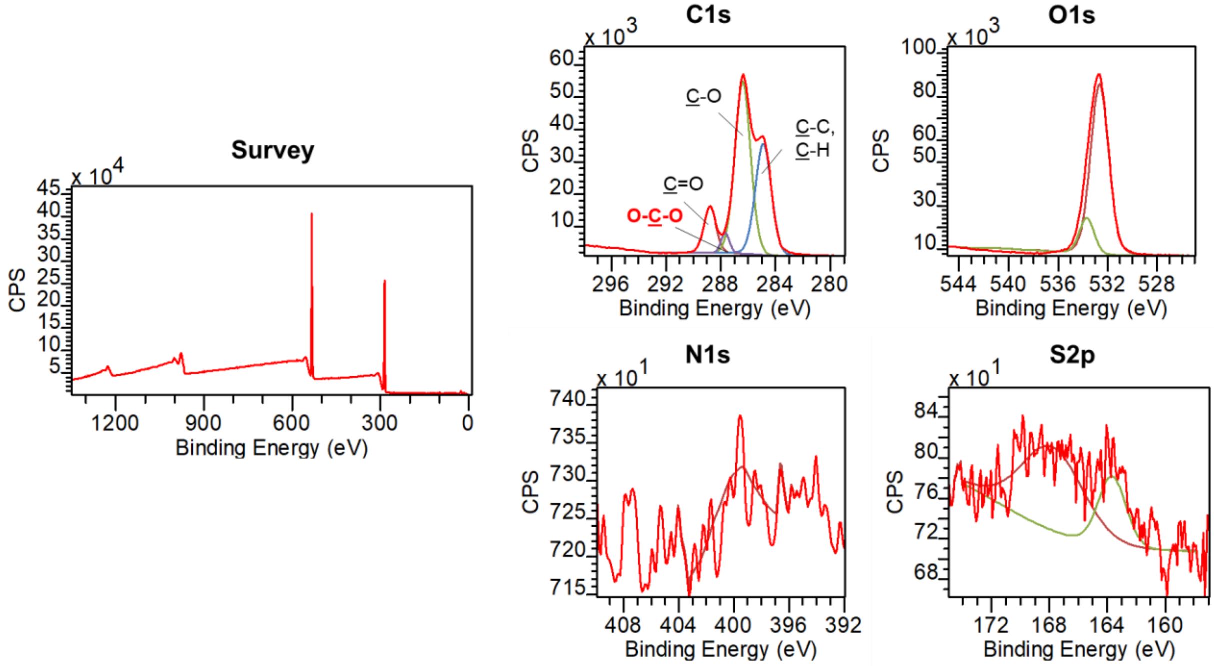

Figure S22. XPS survey and high resolution C1s, O1s, N1s, and S2p, spectra of mannose-SH functionalized G1-containing brush.

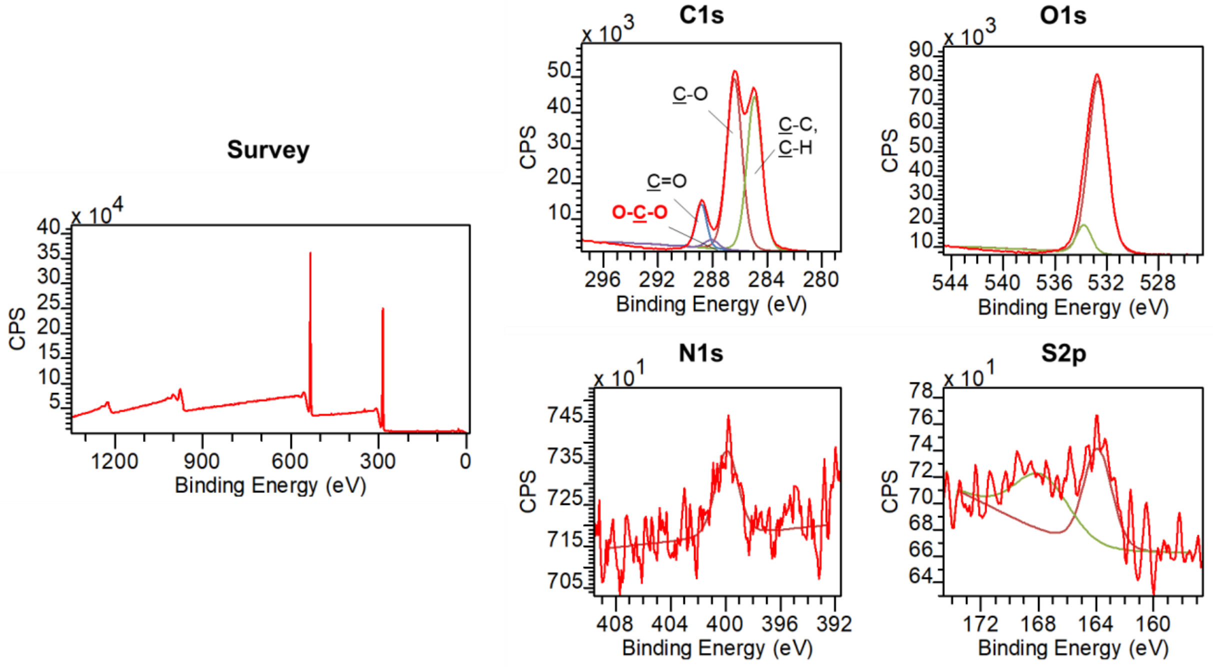

Figure S23. XPS survey and high resolution C1s, O1s, N1s, and S2p, spectra of mannose-SH functionalized G1/AIBN-containing brush.

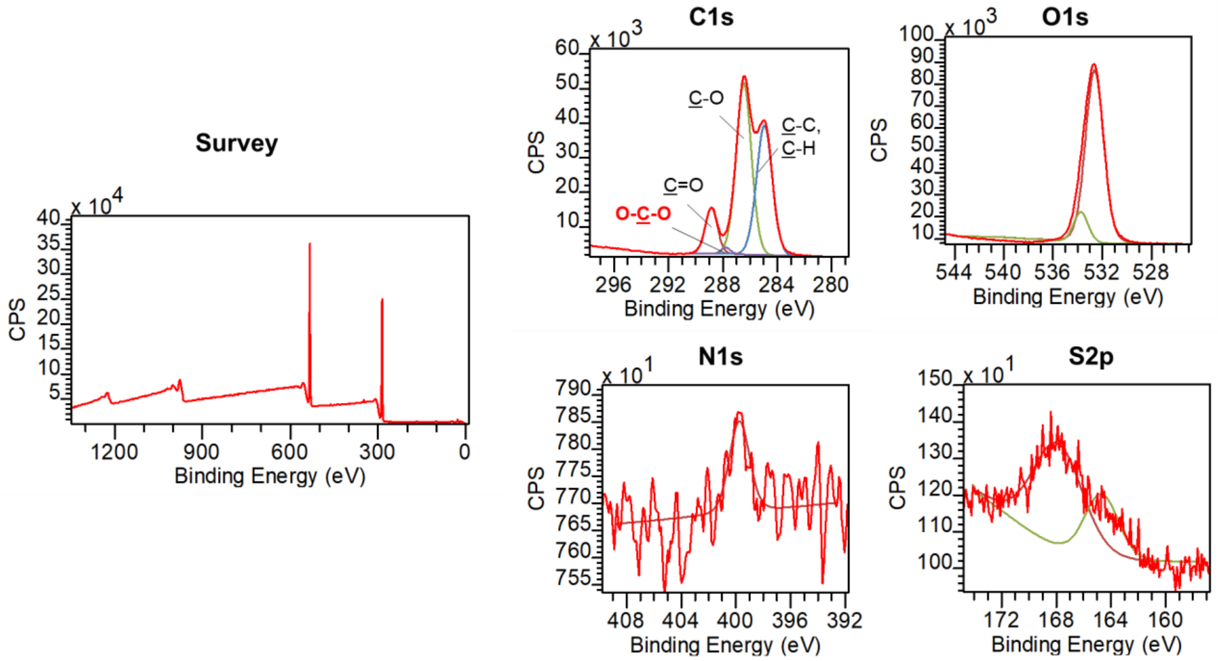

Figure S24. XPS survey and high resolution C1s, O1s, N1s, and S2p, spectra of mannose-SH functionalized G2/AIBN-containing brush.

FTIR Spectra

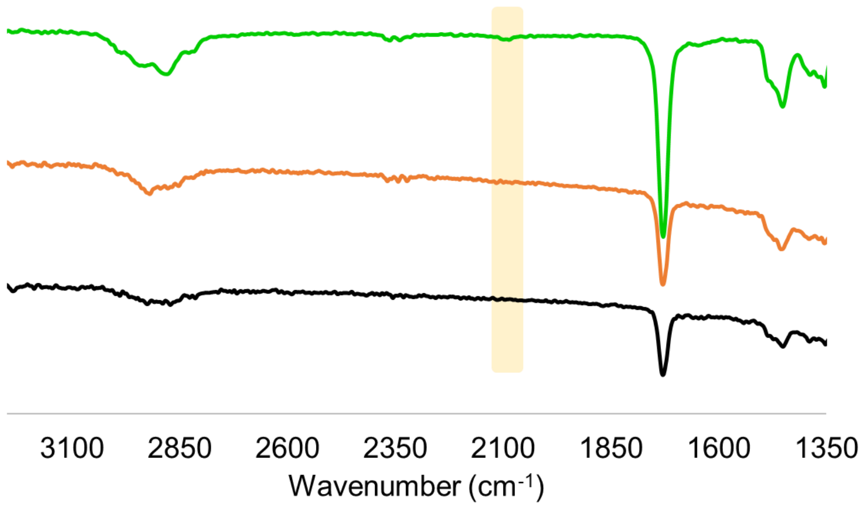

Figure S25. FT-IR spectra of azide functionalized DEGMA containing brushes (green line), BODIPY-alkyne functionalized DEGMA containing brushes (orange line) and Rhodamine conjugated DBCO alkyne functionalized DEGMA containing brushes (black line).

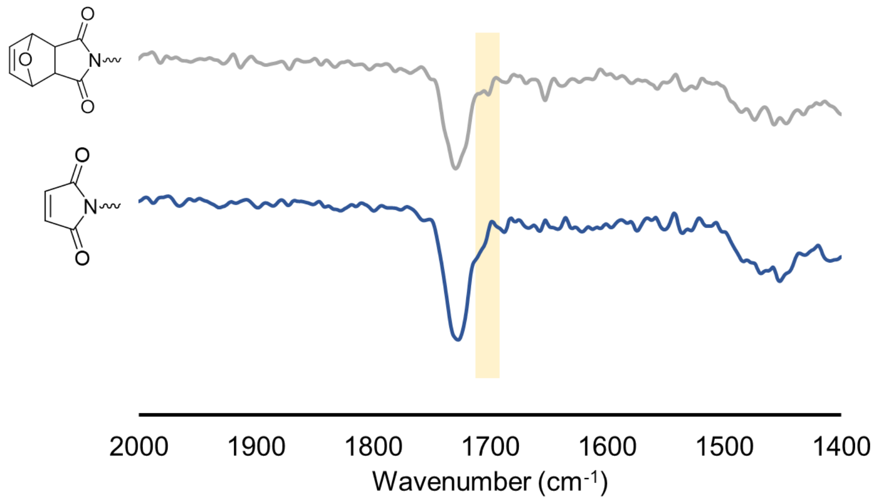

Figure S26. FT-IR spectra of furan-protected maleimide functionalized DEGMA brushes (grey line), and maleimide functionalized DEGMA brushes after retro Diels-Alder reaction (dark blue line).

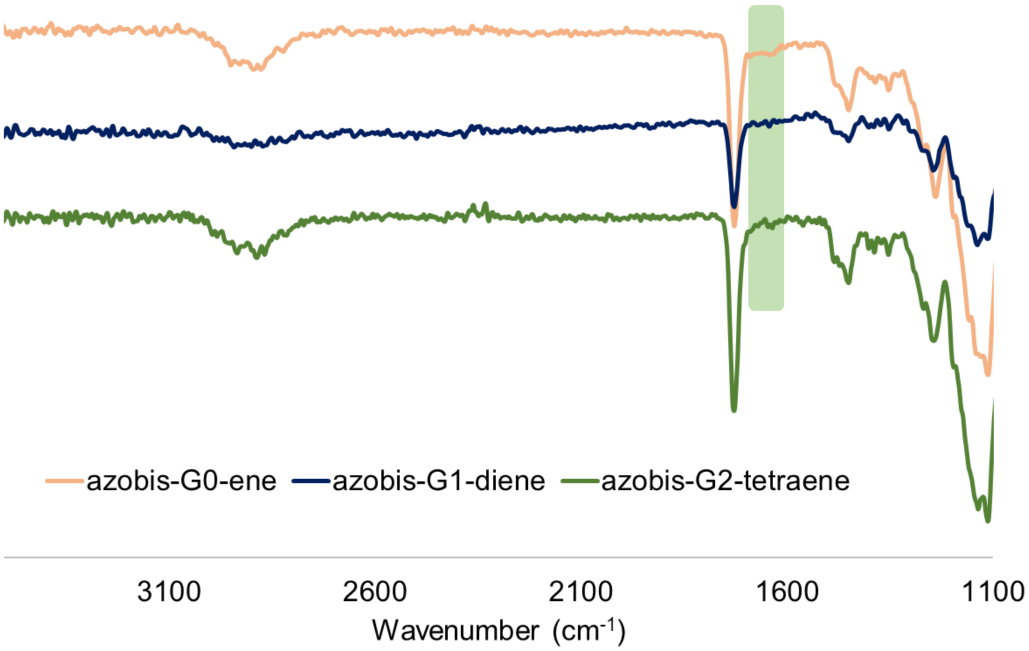

Figure S27. FT-IR spectra of azobis-G0-ene functionalized DEGMA brushes (peach line), azobis-G1-diene functionalized DEGMA brushes (navy blue line), and azobis-G2-tetraene functionalized DEGMA brushes (green line).

Calculation of Grafting Density

To determine number-average molecular weight (M_n), surface RAFT polymerization was carried out in the presence of free CTA and equimolar ratio of [AIBN]/[CTA]. After 6 h polymerization, M_n value was determined as 50 000 g/mol via SEC analysis (M_w/M_n = 1.60). The grafting density (σ, chains/ nm^2) of DEGMA polymer brush were calculated from AFM thickness (*h*, nm) and number-average molecular weight (M_n, g/mol) values using eq 1.[1]

$$\sigma = \frac{h\rho N_A}{Mn \times 10^{21}} \quad (1)$$

where ρ (1.02 g/cm^3) is the density of polymer and N_A (6.02 x 10^{23} mol^{-1}) is the Avogadro number.

References:

(1) Luzinov, I.; Julthongpiput, D.; Malz, H.; Pionteck, J.; Tsukruk, V. V. Polystyrene Layers Grafted to Epoxy-Modified Silicon Surfaces. *Macromolecules* **2000**, *33*, 1043–1048.
